# Hyperbaric Oxygen Regulates Tumor pH to Boost Copper‐Doped Hydroxyethyl Starch Conjugate Nanoparticles Against Cancer Stem Cells

**DOI:** 10.1002/EXP.20240080

**Published:** 2025-04-03

**Authors:** Qingyuan Deng, Ao Hua, Shiyou Li, Zhijie Zhang, Xiang Chen, Qiang Wang, Xing Wang, Zhiqin Chu, Xiangliang Yang, Zifu Li

**Affiliations:** ^1^ Department of Nanomedicine and Biopharmaceuticals College of Life Science and Technology Huazhong University of Science and Technology Wuhan P. R. China; ^2^ Department of Electrical and Electronic Engineering The University of Hong Kong Hong Kong P. R. China; ^3^ School of Biomedical Sciences The University of Hong Kong Hong Kong P. R. China; ^4^ National Engineering Research Center for Nanomedicine Huazhong University of Science and Technology Wuhan P. R. China; ^5^ Key Laboratory of Molecular Biophysics of Ministry of Education Huazhong University of Science and Technology Wuhan P. R. China; ^6^ Hubei Key Laboratory of Bioinorganic Chemistry and Materia Medical Huazhong University of Science and Technology Wuhan P. R. China; ^7^ Hubei Engineering Research Center for Biomaterials and Medical Protective Materials Huazhong University of Science and Technology Wuhan P. R. China

**Keywords:** cancer stem cells, carbonic anhydrase 9, hyperbaric oxygen, nanomedicine, tumor pH

## Abstract

An extracellular acidic environment and an intracellular mildly alkaline environment induced by carbonic anhydrase 9 (CA9) play a critical role in self‐renewal, invasion, migration, and drug resistance of cancer stem cells (CSCs) within hypoxic solid tumors. Here, we report an antitumor strategy leveraging hyperbaric oxygen therapy (HBO) to regulate tumor pH and boost hydroxyethyl starch‐doxorubicin‐copper nanoparticles (HHD‐Cu NPs) against CSCs. HBO overcomes tumor hypoxia, downregulates pH‐regulatory proteins such as CA9, and leads to intracellular accumulation of acidic metabolites. As a result, HBO promotes intracellular acidification of both tumor cells and CSCs, triggering efficient doxorubicin release and the potent copper‐mediated chemical dynamic effect of subsequently administered dual‐acid‐responsive HHD‐Cu NPs. The combination of HBO with HHD‐Cu NPs not only eliminates tumor cells but also inhibits CSCs, altogether leading to potent tumor inhibition. This study explores a new function of clinical‐widely used HBO and establishes a novel combination therapy for treating CSCs abundant hypoxic solid tumors.

## Graphical abstract

Hypoxia and acidic microenvironments are characteristic features of solid tumors, driving tumor growth, drug resistance, and metastasis. To address these issues, we combined hyperbaric oxygen therapy with rational designed hydroxyethyl starch‐doxorubicin‐copper nanoparticles to modulate the acidic microenvironment of tumors while enhancing the efficacy of chemotherapy and chemodynamic therapy to effectively kill hypoxic solid tumors. Our work reveals that alleviating hypoxia and modulating the tumor pH environment are effective for tumor therapy and expected to enhance the efficacy of multiple therapies in the treatment of solid tumors.

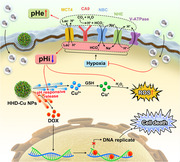

## 1 Introduction

The acidification of the tumor microenvironment is a prominent characteristic of solid tumors [1, 2]. Under hypoxic conditions, the accumulation of hypoxia‐inducible factor 1α (HIF‐1α) promotes the expression of carbonic anhydrase 9 (CA9) [3], a member of the carbonic anhydrase family, which selectively expresses on the surface of tumor cells and gastrointestinal epithelial cells [4]. CA9 catalyzes the reversible hydration of carbon dioxide, coordinating with sodium hydrogen exchanger 1 (NHE1), monocarboxylate transporter 4 (MCT4), sodium bicarbonate cotransporter 1 (NBC), and vacuolar‐type ATPase (V‐ATPase) to regulate proton extrusion from cells while transporting bicarbonate ions into cells [5], preventing the accumulation of acidic metabolites to maintain an mildly alkaline intracellular pH (pHi) and induce an acidic extracellular pH (pHe) [6, 7, 8]. Additionally, CA9 has been correlated with several cancer stem cell (CSC) markers in numerous studies [9, 10, 11]. CSCs, widely blamed as the primary cause of tumor recurrence, metastasis, and treatment resistance [12, 13], exhibit higher CA9 expression and stronger survival capabilities in dysregulated tumor acidic pH than bulk cancer cells. CA9‐mediated dysregulation of tumor pH promotes tumor progression through various mechanisms, including the induction of immune suppression [14], reduced intercellular adhesion [5], enhanced tumor cell proliferation [15, 16], and promotion of self‐renewal and stemness of CSCs [17]. Therefore, regulating tumor pH holds tremendous potential for cancer therapy.

Many researches focus on targeting tumor acidic pHe [18, 19], while normalizing tumor pH also holds promising prospects in cancer treatments [6]. Many therapies regulating tumor pH utilize small compounds or nanoparticles to suppress pH regulators like CA9, NHE1, among others, amplifying the effectiveness of various treatments including chemotherapy [2], radiotherapy [10], photodynamic therapy (PDT) [20], and chemodynamic therapy (CDT) [21, 22]. PDT utilizes photosensitizers under light exposure to produce reactive oxygen species (ROS), which exert cytotoxic effects [23, 24]. Meanwhile, CDT employs metal ions like Fe^2+^ or Cu^2+^ under acidic conditions to convert the tumor endogenous H_2_O_2_ into •OH, eliminating tumor cells [25]. As both CDT and PDT utilize ROS as their cytotoxic mediators, targeting CSCs that are more sensitive to oxidative stress [26] through CA9 inhibitors can achieve outstanding cytotoxic effects [27]. Although regulating pH improves the sensitivity of tumor cells to numerous therapies, there are some limitations. First, CA9 also express in the normal gastrointestinal cells [4], leading to unavoidable off‐target side effects. Moreover, PDT is limited by light penetration depth and oxygen supply [23]. Similarly, CDT effectiveness depends on tumor acidity and the concentration of H_2_O_2_ [28]. Therefore, overcoming hypoxia as well as regulating tumor pH could potentially enhance both CDT and PDT. To illustrate, Lin et al. developed an integrated nanoplatform that explored this strategy [29]. However, alleviating hypoxia and modulating tumor pH in a simple and efficient manner is urgently desirable.

We have previously leveraged hyperbaric oxygen therapy (HBO) as an effective means to alleviate tumor hypoxia [30, 31, 32]. HBO involves breathing pure oxygen under conditions exceeding atmospheric pressure, increasing the concentration of dissolved oxygen in plasma directly [33]. Given that CA9 is induced by hypoxia, we hypothesize that HBO has the capability to downregulate pH‐regulatory proteins such as CA9, MCT4, NHE1, and others, thereby normalizing tumor pH and augmenting tumor therapies. Moreover, HBO treatment can boost nanomedicines delivery efficiency while alleviating severe adverse effects [30, 31, 32, 34]. Although HBO itself cannot kill tumors [30], it can alleviate hypoxia, enhance the delivery of nanomedicines, and regulate tumor pH, boosting nanomedicines involving CDT, PDT, and other therapies.

**FIGURE 1:**
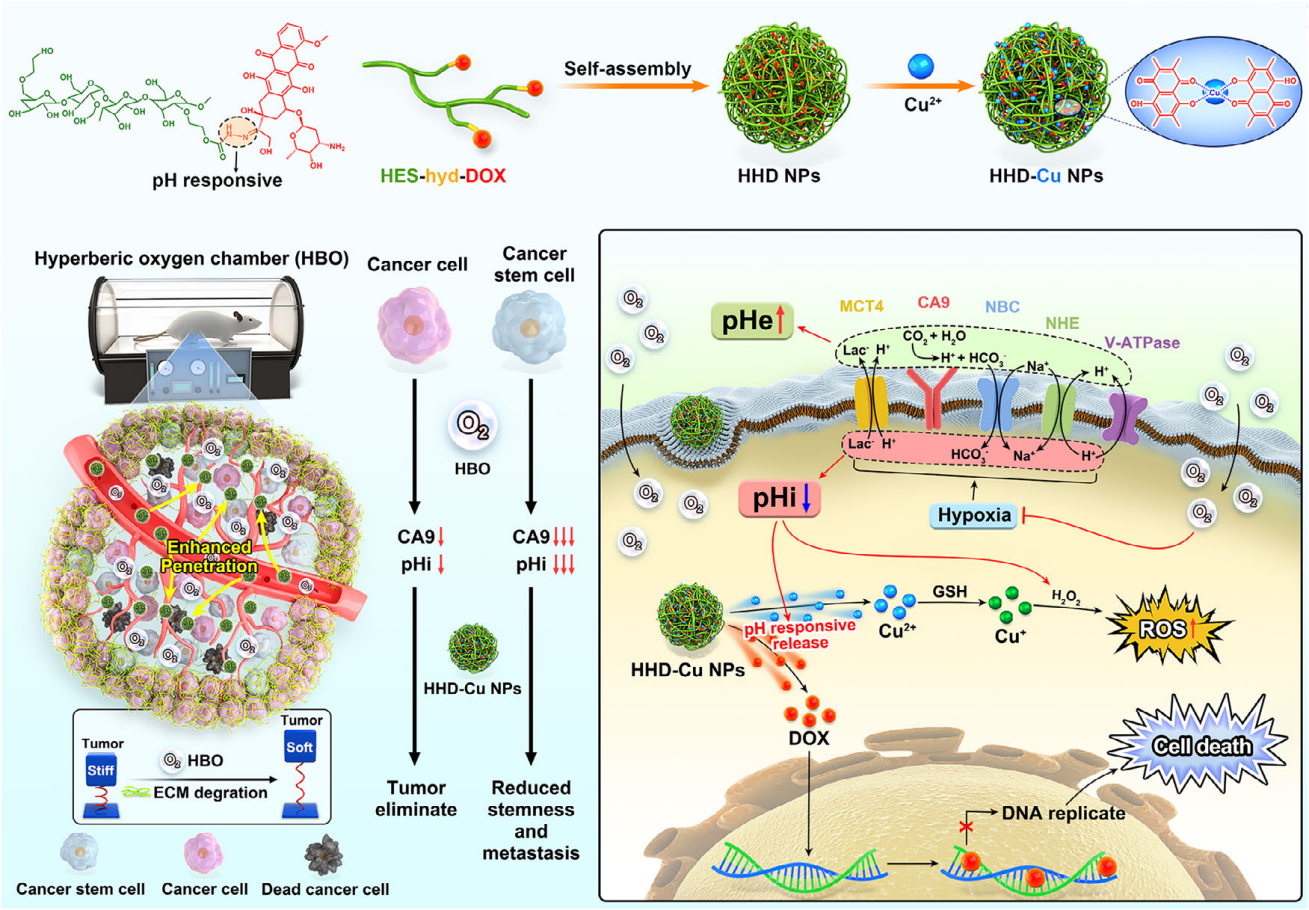
Schematic illustration of HBO regulating tumor pH, reducing stiffness, and enhancing cytotoxicity of HHD‐Cu NPs against both tumor cells and CSCs. Initially, we link DOX to HES utilizing pH‐responsive hydrazone bonds. Then, ultrasonic emulsification and Cu^2+^ are doped onto HHD to form HHD‐Cu NPs. HBO not only regulates tumor pH through reducing CA9 expression but also reduces tumor stiffness to boost nanoparticles tumor accumulation and enrichment. Subsequently, HHD‐Cu NPs release DOX by breaking pH‐responsive hydrazone bonds intracellularly, meanwhile, Cu^2+^ catalyzes endogenous H_2_O_2_ to generate lethal •OH under acidic condition, effectively eliminating both cancer cells and CSCs.

In this study, we first reveal the unique mechanism by which HBO inhibits CA9 and acidifies the pHi of both tumor cells and CSCs. Based on the mechanistic studies, we designed and synthesized dual‐pH‐responsive hydroxyethyl starch‐hydrazone‐doxorubicin‐copper polymer nanoparticles (HHD‐Cu NPs). Doxorubicin (DOX) is linked to HES via pH‐sensitive hydrazone bonds to form HHD, which is then emulsified to generate nanoparticles (HHD NPs). Subsequently, Cu^2+^ is loaded via the coordination interaction between DOX and Cu^2+^, ultimately resulting in the formation of HHD‐Cu NPs. Primarily, HHD‐Cu NPs passively target tumor sites via the enhanced permeability and retention (EPR) effect [35], and HBO can enhance the delivery and accumulation of HHD‐Cu NPs. After HBO treatment, intracellular acidification and extracellular alkalinization not only prevent DOX leakage outside cells but also increase pH‐responsive hydrazone bond (hyd) cleavage within tumor cells and CSCs, facilitating DOX release and amplifying copper‐mediated CDT to generate excessive •OH [36]. The chemotherapy effect of DOX, coupled with amplified oxidative stress, markedly impairs tumor cells and CSCs (Figure 1). HBO, a clinically established therapy, offers a simple and effective method to alleviating hypoxia, regulating tumor pH, and enhancing drug delivery. This study establishes a novel treatment paradigm by leveraging HBO to regulate tumor pH and augment chemotherapy as well as CDT against CSCs.

## 2 Results

### 2.1 HBO Overcomes Tumor Hypoxia to Suppress pH‐Regulatory Pathways

**FIGURE 2:**
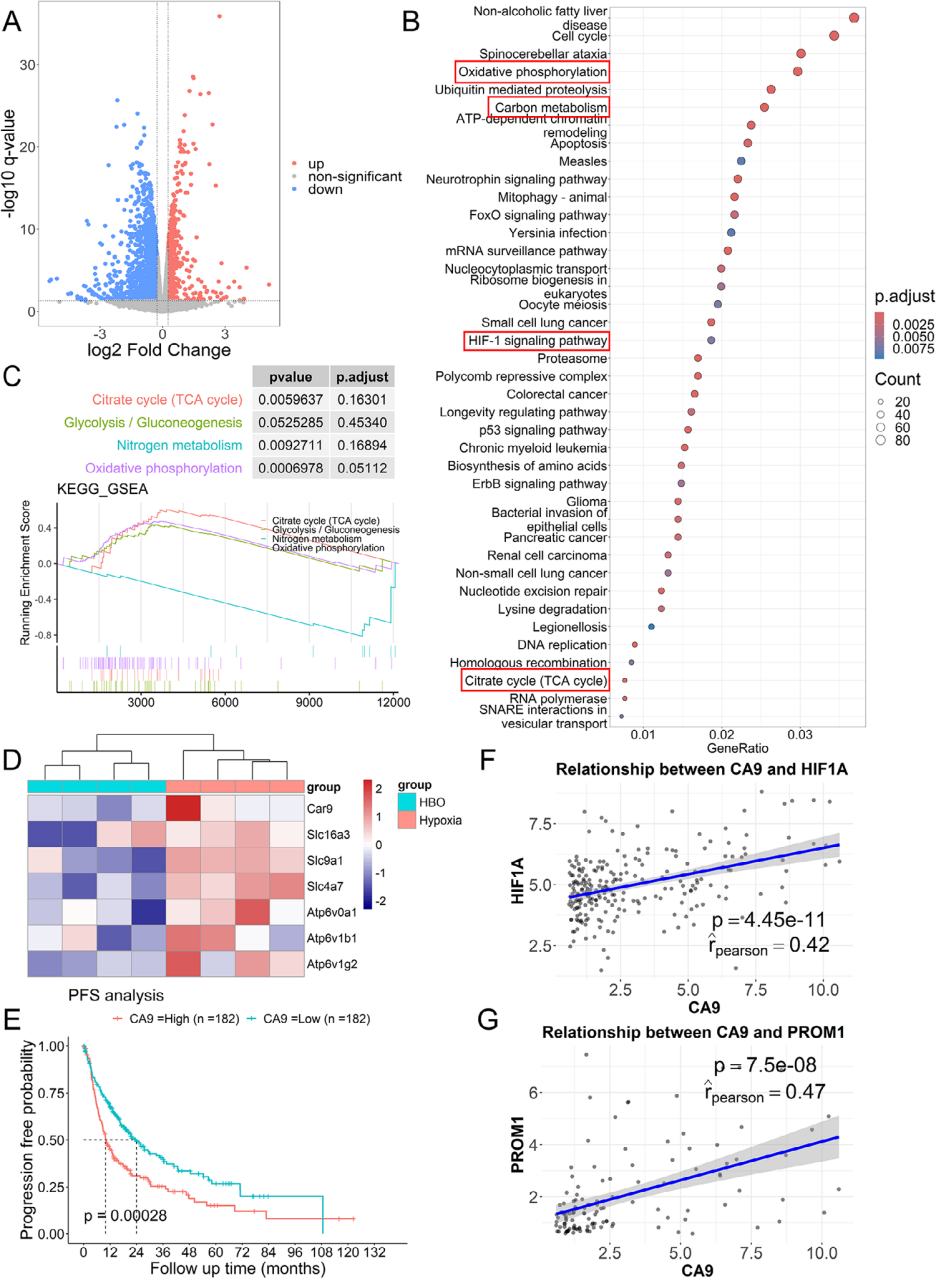
HBO overcomes tumor hypoxia to suppress pH‐regulatory pathways. (A) Volcano plot of differentiated expressed genes between HBO and hypoxia of H22 cancer cells. (B) Significantly changed pathways in KEGG enrichment analysis of H22 cancer cells. (C) GSEA enriched pathways with lowest *p* value and highest absolute value of NES in H22 cancer cells. (D) H22 cancer cells gene transcription heat‐map of CA9 (Car9) and its associated pH regulatory genes, including MCT4 (Slc16a3), NHE1 (Slc9a1), NBC (Slc4a7), and V‐ATPase (Atp6v0 and Atp6v1). (E) The Kaplan–Meier survival curve for CA9 gene in TCGA‐LIHC database and the follow‐up time cutoff were set at 60 months. (F) Pearson correlation of HIF‐1α and CA9 expression in TCGA‐LIHC database. (G) Pearson correlation of PROM1 and CA9 expression in TCGA‐LIHC database.

To reveal the impact of hyperbaric oxygen (HBO) on tumor cells, we conducted RNA‐seq on H22 liver cancer cell line and divided it into two groups. The control group consisted of H22 cells that were cultured at 1% oxygen for 48 h while the experimental group underwent 1.5 h of HBO treatment after 48 h of 1% oxygen exposure. The transcriptome sequencing yielded expression data of 15,116 genes, and differential expression analysis using edgeR [37] identified 2364 differentially expressed genes (|log2FC| > 0.263, FDR ≤ 0.05), as illustrated in a volcano plot in Figure 2A. Subsequently, Kyoto Encyclopedia of Genes and Genomes (KEGG) enrichment analysis and gene ontology (GO) enrichment analysis were performed on differentially expressed genes by clusterProfiler [38]. KEGG analysis identified 40 significantly altered pathways, as displayed in Figure 2B, while GO analysis demonstrated the top ten differentially expressed pathways in biological processes (BP), cellular components (CC), and molecular functions (MF), which are presented in Figure S1. KEGG enrichment analysis revealed significant changes in pathways associated with energy metabolism, such as glycolysis, tricarboxylic acid (TCA) cycle (Figure S2), and oxidative phosphorylation (OXPHOS) (Figure S3). HIF‐1α pathway also exhibited significant change. Figure 2C displays gene set enrichment analysis (GSEA) results and presents four pathways with high normalized enrichment score (NES) absolute values and low *p*‐values: TCA cycle, glycolysis, nitrogen metabolism, and OXPHOS. Notably, while some pathways related to energy metabolism showed upregulation, nitrogen metabolism exhibited downregulation. Genes significantly changed in the nitrogen metabolism pathway after HBO treatment were all members of the carbonic anhydrase family, including CA9 (Figure S4). These results indicate that HBO may not only alter the energy metabolism of H22 cells [39] but also affect cellular acid‐base homeostasis. We further analyzed the expressions of CA9 and its related pH‐regulatory genes in Figure 2D. The expression of these genes showed downregulation after HBO treatment, suggesting that HBO might potentially modulate tumor pH.

To validate the clinical relevance of CA9, we analyzed transcriptome data and clinical data of 364 patients from the Cancer Genome Atlas Liver Hepatocellular Carcinoma (TCGA‐LIHC) database. To that end, we plotted progression‐free survival (PFS) curve for the CA9 gene in Figure 2E. It is evident that patients with high CA9 expression had a worse prognosis, with a median survival time reduced by approximately 50% compared to the low CA9 expression group (*p* = 0.0008). Pearson correlation analysis of gene expression in Figure 2F,G reveals a moderate positive correlation between CA9 expression and HIF‐1α and CD133 (PROM1), which is a tumor cell stemness surface marker [40], with Pearson correlation coefficients of 0.42 and 0.47, respectively. Therefore, high CA9 expression indicates severe tumor hypoxia and high tumor stemness.

Together, HBO downregulates the expressions of pH‐regulatory genes such as CA9 in H22 cancer cells, while CA9 is also associated with poor prognosis in liver cancer patients, suggesting that HBO might have the potential to regulate tumor pH and augment cancer treatment efficacy.

### 2.2 Preparation and Characterizations of pH‐Responsive HHD‐Cu NPs

**FIGURE 3:**
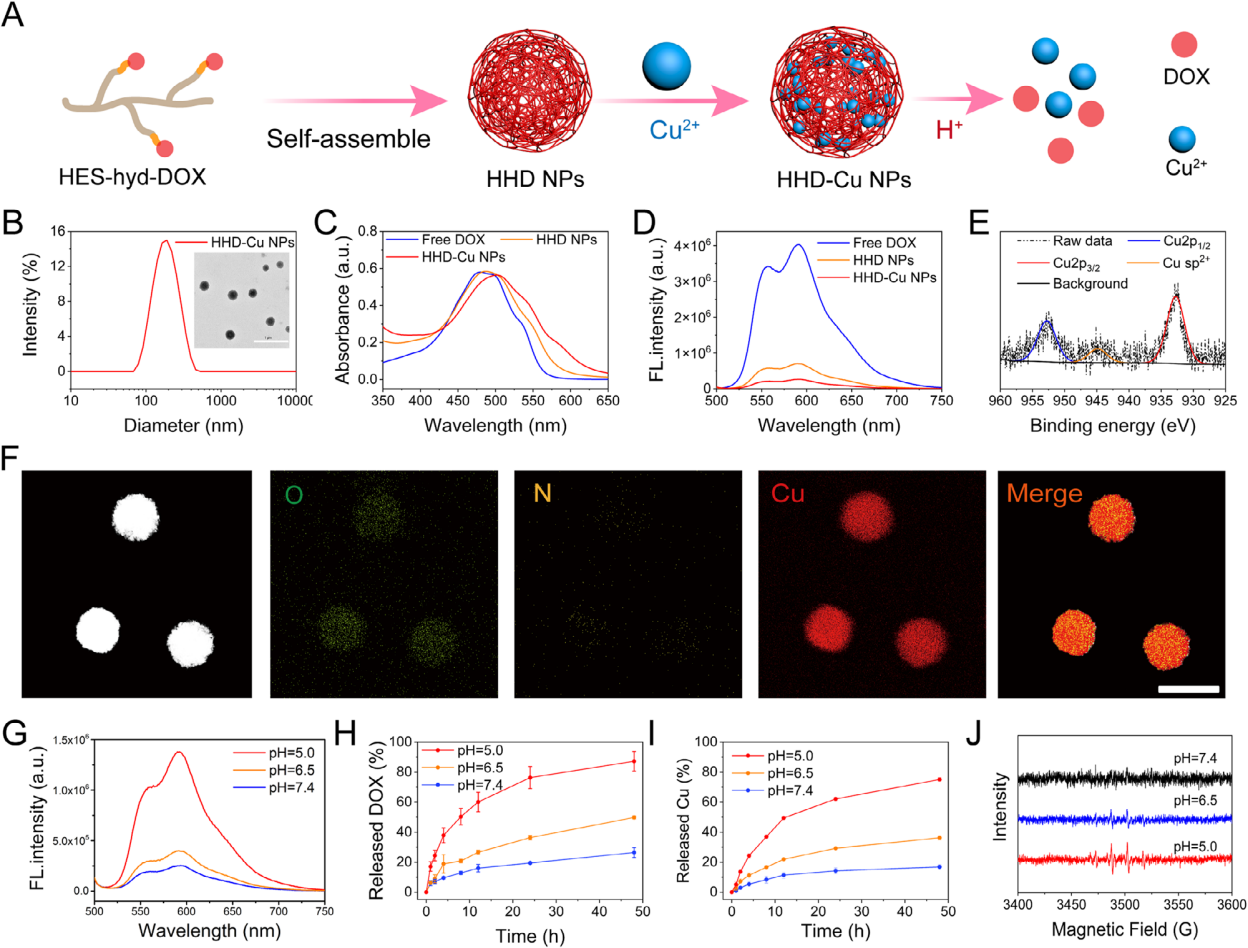
Preparation and characterizations of pH‐responsive HHD‐Cu NPs. (A) The schematic diagram for the preparation of pH‐responsive HHD‐Cu NPs. (B) DLS size distribution and TEM images of HHD‐Cu NPs (Scale bar: 1 µm). (C) The UV–vis absorption spectra of DOX, HHD NPs, and HHD‐Cu NPs. (D) Fluorescence intensity of DOX, HHD NPs, and HHD‐Cu NPs. (E) Survey XPS spectrum of HHD‐Cu NPs. (F) Elemental mappings of HHD‐Cu NPs (Scale bar: 200 nm). (G) Changes in the fluorescence spectra of HHD‐Cu NPs after incubation in different pH buffer solutions (pH 7.4, 6.5, and 5.0). The release profiles of (H) DOX and (I) Cu from HHD‐Cu NPs after incubation in different pH buffer solutions. (J) ESR spectra of ·OH trapped by DMPO.

Based on the result that HBO downregulates pH‐regulatory genes, including CA9, we hypothesized that HBO treatment could lower the intracellular pH of tumor cells to augment pH‐responsive tumor therapies. Therefore, we designed acid‐responsive HHD‐Cu NPs. Figure S5 shows the synthetic route of HES‐hyd‐DOX. Initially, DOX was linked to hydroxyethyl starch through a pH‐responsive hydrazone linker, as confirmed by hydrogen nuclear magnetic resonance (^1^H NMR) in Figure S6, indicating the successful synthesis of HES‐hyd‐DOX. After ultrasonic emulsification, HHD nanoparticles (HHD NPs) self‐assembled, and the addition of copper chloride turned the color from orange red to purple, indicating the coordination of copper ions with DOX. After dialysis in ultra‐pure water for 72 h (changing fresh water every 4 h) and three ultrafiltration cycles using a 100 kDa ultrafiltration membrane, the final HHD‐Cu NPs were obtained. The preparation scheme is illustrated in Figure 3A. The hydrated particle size of HHD‐Cu NPs measured by dynamic light scattering was approximately 180 nm. In transmission electron microscopy (TEM), HHD‐Cu NPs exhibited regular spherical shapes, as shown in Figure 3B. Zeta potential in Figure S7 shows both HHD NPs and HHD‐Cu NPs were approximately 15 mV. HHD‐Cu NPs demonstrated excellent colloidal stability, as shown in Figure S8; nanoparticle size remained around 180 nm, and the PDI were approximately 0.2 in PBS over a 7‐day period. Ultraviolet–visible spectroscopy (UV–vis) measurements in Figure 3C show that the absorption peak of free DOX was at 480 nm, while the absorption peak of HHD NPs slightly shifted to 486 nm, indicating successful loading of DOX. The absorption peak of HHD‐Cu NPs further shifted to 502 nm. The weakened fluorescence of HHD NPs compared to free DOX and the further decrease in fluorescence of HHD‐Cu NPs indicate the successful coordination of Cu with DOX [41] (Figure 3D). The X‐ray photoelectron spectroscopy (XPS) survey in Figure 3E demonstrates the presence of copper ion 2p1/2 and 2p3/2 peaks, along with an intermediate satellite peak in HHD‐Cu NPs, confirming the successful loading of divalent copper ions [42]. Field emission transmission electron microscopy (FTEM) results in Figure 3F illustrate the elemental distribution of HHD‐Cu NPs, confirming a successful copper load.

Furthermore, we examined the release behaviors of HHD‐Cu NPs. First, the fluorescence recovery phenomenon due to hydrazone bond cleavage under acidic conditions was used to track the release of DOX. HHD‐Cu nanoparticles were immersed in PBS at different pH values and incubated at 37°C for 4 h. Fluorescence spectrum measurements in Figure 3G show a slight fluorescence recovery at pH 6.5, with a 59.7% increase in fluorescence intensity at 592 nm compared to that at pH 7.4. At pH 5.0, the fluorescence intensity increased by approximately 450%. These results suggest that the release of DOX from HHD‐Cu NPs exhibits significant acid responsiveness. In different pH PBS solutions containing 0.5% Tween‐80, we tested the release of HHD‐Cu NPs over time and presented the results in Figure 3H,I. With increased acidity, both DOX and copper ion release from HHD‐Cu NPs increased. At the time point of 48 h, released DOX increased from 26.4% at pH 7.4 to 49.8% at pH 6.5 and to 83.2% at pH 5.0, respectively. The released copper ion also increased from 12.6% at pH 7.4 to 33.4% at pH 6.5 and to 75.0% at pH 5.0. Subsequently, we used electron spin resonance (ESR) to test hydroxyl radicals generated by HHD‐Cu NPs‐mediated CDT in solutions of different pH values. With increasing acidity, the peak height of hydroxyl radicals increased (Figure 3J). MB degradation experiments depicted in Figure S9 also confirms this conclusion, indicating an acidity‐dependent generation of hydroxyl radicals.

A series of experiments indicated excellent stability as well as pH‐responsive release and hydroxyl radical‐inducing capability of HHD‐Cu NPs (Figure 3). Considering that HBO inhibits the expressions of pH‐regulatory genes (Figure 2), HBO might augment chemotherapy and CDT effects of pH‐responsive HHD‐Cu NPs by increasing intracellular acidity.

### 2.3 Enhancement of H22 Cell Killing by HBO Disrupted Hypoxia and Lowered pHi

**FIGURE 4:**
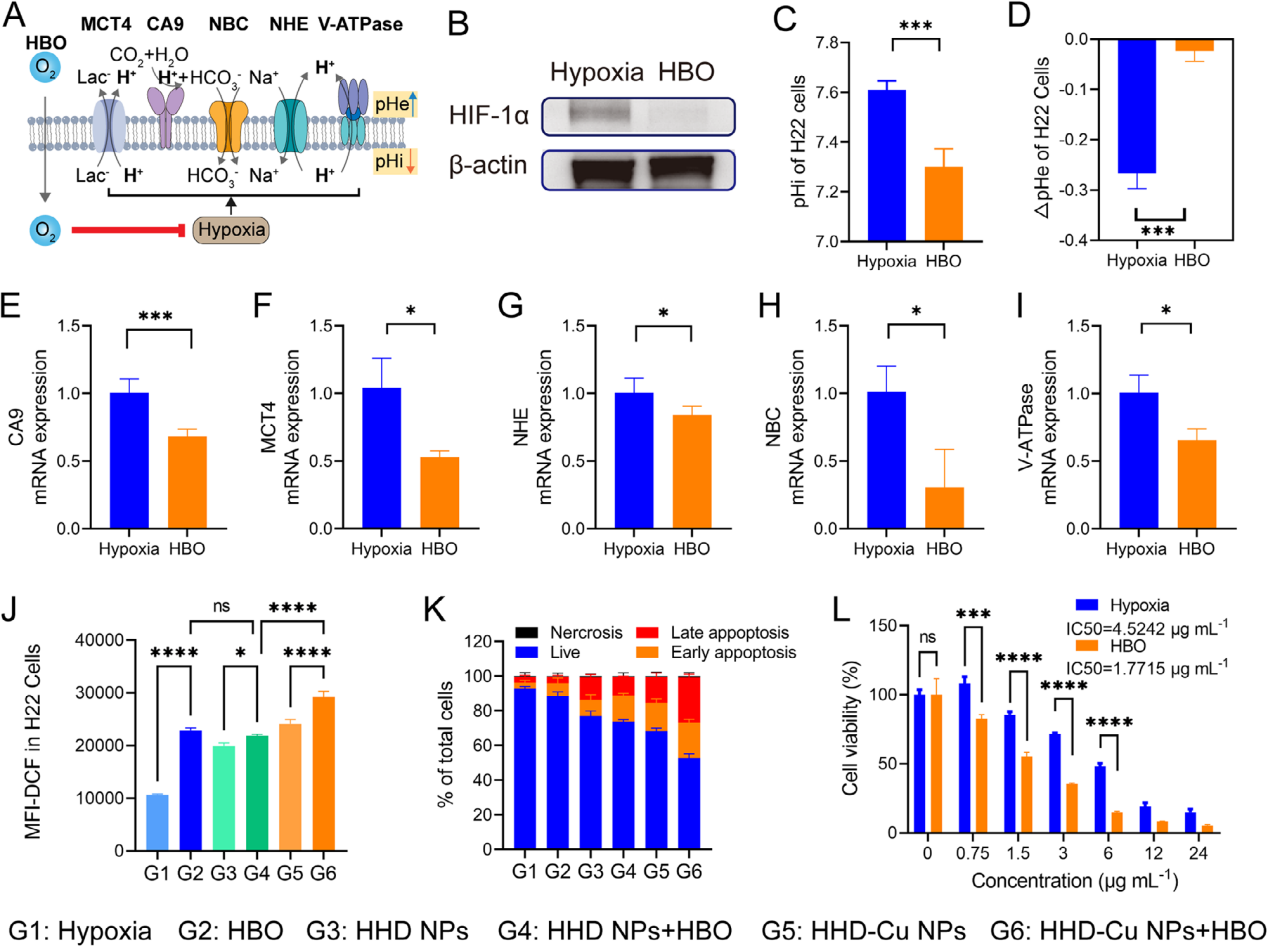
Enhancement of H22 cell killing by HBO disrupted hypoxia and lowered pHi. (A) Schematic diagram illustrating the regulation of intra‐ and extra‐cellular pH of H22 cancer cells by HBO. (B) Western Blot detection of HIF‐1ɑ expression in H22 cancer cells. (C) Measurements of H22 cancer cells pHi after different treatments. (D) Change of H22 cancer cells pHe after different treatments. (E–I) Regulation of transcriptional level expression of genes (CA9, MCT4, NHE, NBC, and V‐ATPase) related to pH‐regulatory pathways of H22 cancer cells. (J) ROS levels in H22 cancer cells after different treatments identified by flow cytometry. (K) Quantitation of cell apoptosis results measured by flow cytometry. (L) Cell viability assay of HHD‐Cu NPs on H22 cancer cells. Statistical significance was calculated by unpaired two‐sided Student's *t‐*test or One‐way ANOVA followed by post hoc Tukey's test and represented as the mean ±SEM. *p* values: *p < 0.05, **p < 0.01, ***p < 0.001, ****p < 0.0001, ns stands for not significant.

Inspired by the transcriptome findings in Figure 2, we performed western blot and qPCR experiments to investigate the effects of HBO on overcoming hypoxia and reducing expressions of pH‐regulatory genes (Figure 4A). First, Figure 4B and Figure S10 demonstrate that HBO treated group exhibited a significant decrease in expressions of HIF‐1α and CA9 proteins in H22 cells relative to the control group. Subsequently, we used the BCECF‐AM probe to detect pHi. The probe is hydrolyzed and fluoresces only within cells. The fluorescence intensity ratio between 490 nm excitation/535 nm emission (F490) and 440 nm excitation/535 nm emission (F440) increases linearly with increasing pH between 5 and 9. pHi of H22 cells was quantified according to the previously reported method [2]. Initially, H22 cells were treated with nigericin to balance intracellular pH with HHBS buffer at different external pH values. The ratio of F490/F440, determined by measuring fluorescence intensity of F490 and F440 at each pH, was plotted against pH to produce a linear standard curve, as exhibited in Figure S11. The pHi is depicted in Figure 4C, which shows the intracellular pH of H22 cells in the hypoxia group is approximately 7.6; after HBO treatment, pHi decreases to around 7.3, indicating an approximate 0.3 reduction by HBO. Meanwhile, we measured extracellular pH changes within 24 h post‐HBO treatment and hypoxic conditions using a pH meter. Figure 4D illustrates that hypoxia significantly reduced extracellular pH by approximately 0.26. In contrast, there was no significant change in pHe of HBO treated group. Furthermore, qPCR data presented in Figure 4E–I reveals that HBO treatment induced a significant reduction in mRNA expressions of CA9 (Car9), MCT4 (Slc16a3), NHE1 (Slc9a1), NBC (Slc4a7), and V‐ATPase (Atpv1g2).

Since HBO significantly decreases pH‐regulatory genes and proteins, it is reasonable to speculate that HBO would augment the therapeutic efficacy of pH‐responsive HHD‐Cu NPs. To that end, we measured intracellular ROS and cell apoptosis. First, we cultured H22 cells and divided them into six groups, including hypoxia (G1), HBO (G2), HHD NPs (G3), HHD NPs + HBO (G4), HHD‐Cu NPs (G5), and HHD‐Cu NPs + HBO (G6). Intracellular ROS was detected using a DCFH‐DA probe, and the mean fluorescence intensity (MFI) of DCF was measured using a flow cytometer and presented in Figure 4J. Comparatively, groups with HBO treatment (G2, G4, and G6) exhibited a significant increase in ROS compared to the corresponding groups without HBO (G1, G3, and G5). Besides, groups with copper‐containing HHD‐Cu NPs (G5 and G6) showed a significant increase in ROS compared to HHD NPs groups (G3 and G4). Similarly, HHD‐Cu NPs + HBO group (G6) showed a significant increase in ROS compared to HHD NPs + HBO group (G4). Crucially, MFI‐DCF of HHD‐Cu NPs + HBO group (G6) increased by approximately 174.4% compared to hypoxia group (G1), indicating that HHD‐Cu NPs combined with HBO can significantly trigger ROS generation within H22 cells. These results also indicate that both HBO and copper in HHD‐Cu NPs are essential to ROS generation. However, the generated ROS could be counteracted by the reductive substance *N*‐acetyl‐L‐cysteine (NAC), as seen in Figure S12. Next, Annexin V/7‐AAD staining was employed to detect apoptosis in H22 cells. Figure 4K and Figure S13 indicate that HBO treated groups (G2, G4, and G6) exhibit an increase in overall apoptosis ratio compared to corresponding groups without HBO (G1, G3, and G5), while copper‐doped groups (G5 and G6) showed an increased apoptosis compared to groups without copper (G3 and G4). Consistent with ROS generation, HBO promotes cell apoptosis induced by HHD‐Cu NPs. Subsequent cell viability experiments on H22 cells were conducted and presented in Figure 4L, which revealed that the half maximum inhibition concentration (IC50) of HHD‐Cu NPs decreased from 4.5242 µg mL^−1^ in hypoxia group to 1.7715 µg mL^−1^ in HBO group, showing a significant enhancement of HHD‐Cu cytotoxicity by HBO. It is worth noting that HBO did not cause changes in pHi or ROS level in HUVEC cells, while HHD‐Cu NPs showed no toxicity on HUVEC cells under a concentration of 5 µg mL^−1^ (Figure S32). These results indicated that the combination of HBO and HHD‐Cu NPs showed almost no killing effect on normal cells and exhibited a good safety profile.

In summary, HBO treatment led to a decrease in transcription and protein expression of pH‐regulatory proteins like CA9 in H22 cells, resulting in a decrease in pHi, which enhanced HHD‐Cu NPs‐induced ROS generation to trigger apoptosis in H22 cells. As such, HBO augments HHD‐Cu NPs cytotoxicity against H22 cancer cells.

### 2.4 HBO Boosts HHD‐Cu NPs on CSCs Elimination and Stemness Suppression

**FIGURE 5:**
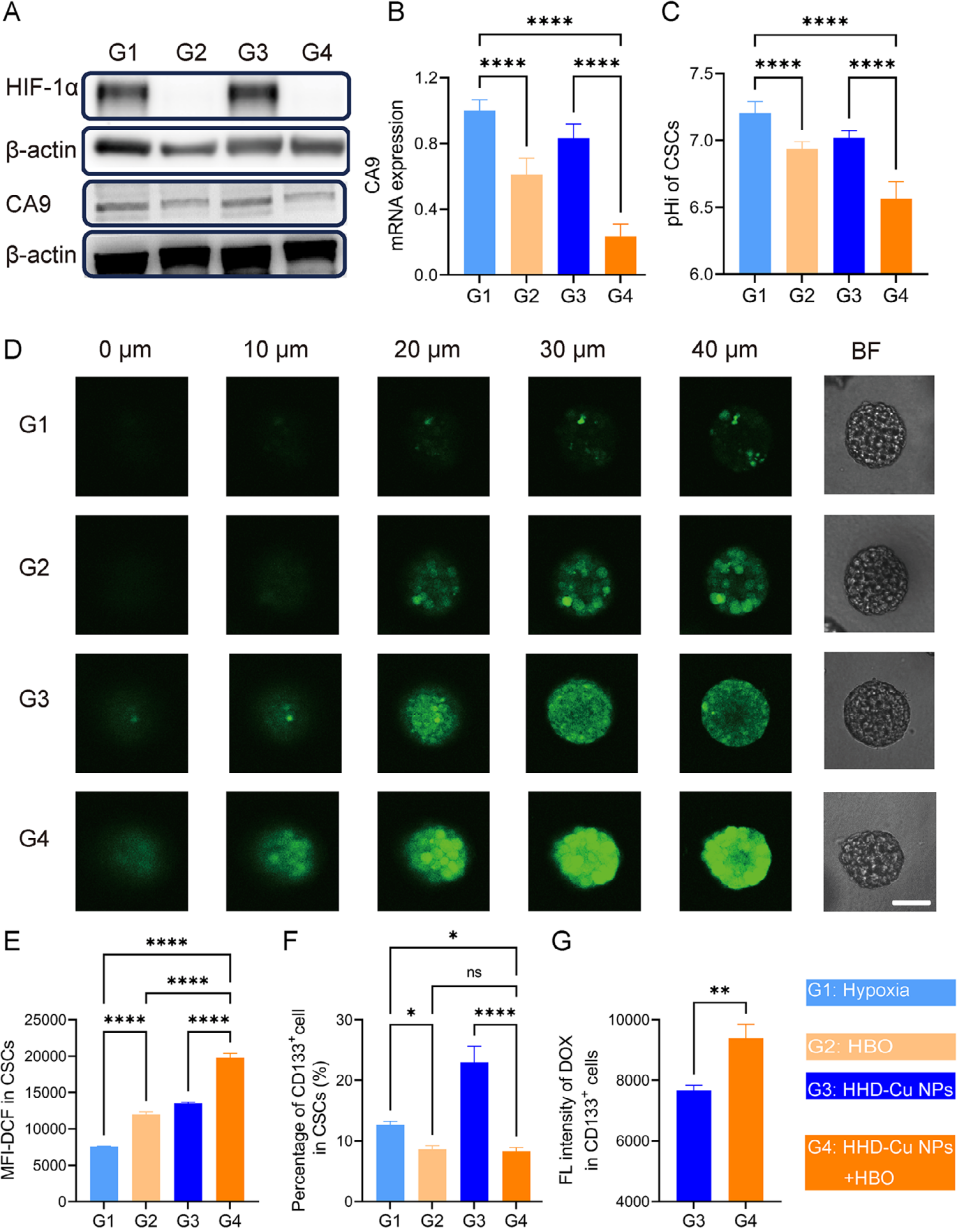
HBO boost HHD‐Cu NPs on CSCs elimination and stemness suppression. (A) Western Blot detection of HIF‐1ɑ and CA9 expression in CSCs. (B) qPCR analyses of CA9 mRNA levels of CSCs after various treatments. (C) Measurements of H22 CSCs pHi after different treatments. ROS levels in CSCs after different treatments identified by CLSM (D) and flow cytometry (E). (F) Percentage of CD133^+^ in H22 CSCs after different treatments characterized by flow cytometry. (G) Uptake of HHD‐Cu NPs by CSCs. Statistical significance was calculated by unpaired two‐sided Student's *t* test or One‐way ANOVA followed by post hoc Tukey's test and represented as the mean ±SEM. *p* values: *p < 0.05, **p < 0.01, ***p < 0.001, ****p < 0.0001, ns stands for not significant.

Numerous studies indicate a correlation between hypoxia‐related proteins like HIF‐1α and CA9 as well as the stemness of tumor cells [17, 43, 44]. Therefore, we specifically investigated the effects of HBO on H22 CSCs. Utilizing CSCs selected via 3D fibrin gels [45], we obtained H22 CSCs with elevated expression of stemness marker CD133 (Figure S14). We also confirmed that the expression of CA9 was higher in CSCs than in H22 cells (Figure S15). We hypothesized that HBO's improvement in CSCs killing could be caused by a significant decrease in pH‐regulatory gene expression and extracellular pH. H22 CSCs were divided into four groups including hypoxia (G1), HBO (G2), HHD‐Cu NPs (G3), and HHD‐Cu NPs + HBO (G4). First, we validated that HBO treatment significantly downregulated proteins of HIF‐1α and CA9 in H22 CSCs (Figure 5A). Assessment of CA9 mRNA transcription in different groups showed that HBO significantly inhibited CA9 mRNA transcription (Figure 5B). Specifically, CA9 expression was significantly reduced in all HBO‐treated groups (G2 and G4) compared to hypoxia groups (G1 and G3). Ultimately, CA9 mRNA in HHD‐Cu NPs + HBO group (G4) decreased to 23.7% of the hypoxia group (G1). Similar to H22 cells, we verified whether HBO treatment directly caused changes in pHi and pHe of H22 CSCs. Figure 5C shows quantification of pHi using the BCECF‐AM probe. HBO group (G2) exhibited a pHi decrease of about 0.3 compared to hypoxia group (G1), while HHD‐Cu NPs + HBO group (G4) showed a more pronounced decrease of about 0.6 compared to hypoxia group (G1), reaching a pH as low as 6.6. We further examined pHe of H22 CSCs using a pH meter (Figure S16); HBO significantly suppressed extracellular acidification. Within 24 h, pHe decreased by 0.55 in the hypoxia group, whereas in the HBO‐treated group, pHe decreased by only about 0.2. The pHi reduction can activate intracellular Fenton reactions [46] and favors drug release from pH‐responsive nanoparticles (Figure 3H,I). Therefore, we believe that HBO could enhance HHD‐Cu NPs cytotoxicity against CSCs.

Next, we tested the killing effect of HBO and HHD‐Cu on H22 CSCs. Firstly, we examined HHD‐Cu NPs induced ROS. Using confocal laser scanning microscopy (CLSM) to observe DCFH‐DA‐stained H22 3D tumor spheres, Figure 5D illustrates that fluorescence intensity significantly increased in HHD‐Cu NPs + HBO group (G4) compared to hypoxia group (G1), due to prominent ROS generation. There was a slight increase in the fluorescence intensity in HBO group (G2) and HHD‐Cu NPs group (G3) than G1. But the fluorescence was more concentrated in the periphery region of 3D tumor spheres for these two groups. Additionally, we quantified ROS in H22 CSCs using flow cytometry. According to Figure 5E, HHD‐Cu NPs + HBO (G4) triggered an increase of about 170% in ROS content compared to the hypoxia group (G1), whereas the HBO group (G2) and HHD‐Cu NPs group (G3) exhibited moderate enhancements. The cytotoxicity experiment result in Figure S17 confirms that HBO significantly reduced IC50 of HHD‐Cu NPs on H22 CSCs, decreasing from 0.8514 µg mL^−1^ without HBO to 0.4477 µg mL^−1^ with HBO. HBO demonstrates a significant enhancement on HHD‐Cu NPs cytotoxicity against CSCs. Interestingly, the killing of CSCs by HBO and HHD‐Cu NPs was significantly higher than that of H22 cells, with a nearly four‐fold reduction in IC50, which may be explained by the greater sensitivity of CSCs toward oxidative stress [47, 48]. We also assessed the impact of combination treatments on self‐renewal and proliferation of CSCs via 3D tumor spheres formation assay (Figure S18). The ability of H22 CSCs to form spheres was significantly repressed in HBO‐treated groups (G2, G4, and G6) compared to non‐HBO‐treated groups (G1, G3, and G5). Meanwhile, the size and number of tumor spheres in HHD‐Cu NPs treated groups (G5 and G6) were noticeably reduced compared to HHD NPs treated groups (G3 and G4), indicating that Cu^2+^ mediated CDT contributes to the suppression of stemness. HHD‐Cu NPs + HBO treatment significantly inhibited the number and size of 3D tumor spheres, suggesting that this combination strategy can effectively reduce self‐renewal and proliferation of H22 CSCs.

Since HBO combined with HHD‐Cu NPs can effectively induce ROS and impair CSCs, we further evaluated the impact of combination treatments on CSCs stemness by measuring the expression of stemness marker CD133. Figure 5F and Figure S19 reveal that HBO‐treated groups (G2 and G4) showed a decrease in the proportion of CD133^+^ cells compared to non‐HBO‐treated groups (G1 and G3). However, HHD‐Cu NPs treatment alone increased the proportion of CD133^+^ cells, possibly related to CSCs' resistance to DOX [49]. Notably, the fluorescence intensity of DOX was significantly higher in CD133^+^ CSCs compared to CSCs not treated with HBO (Figure 5G). The reason was presumably ascribed to the fact that HBO lowered CSCs extracellular pH and promoted the release of DOX from pH‐responsive HHD‐Cu NPs. Meanwhile, Figure S33 shows that the uptake of HHD‐Cu NPs by CSCs is 1.6 times more than that of HUVEC cells, indicating that HHD‐Cu NPs accumulate more in CSCs than in normal cells.

Taken together, HBO downregulates transcription and protein expressions of pH regulatory machinery, leading to a significant decrease in intracellular pH of CSCs. Therefore, combination treatments of HBO and HHD‐Cu NPs effectively induce ROS generation, potently suppress H22 CSCs, and significantly reduce their stemness and self‐renewal abilities.

### 2.5 HBO Promotes HHD‐Cu NPs Tumor Targeting Delivery by Depleting Tumor ECM

**FIGURE 6:**
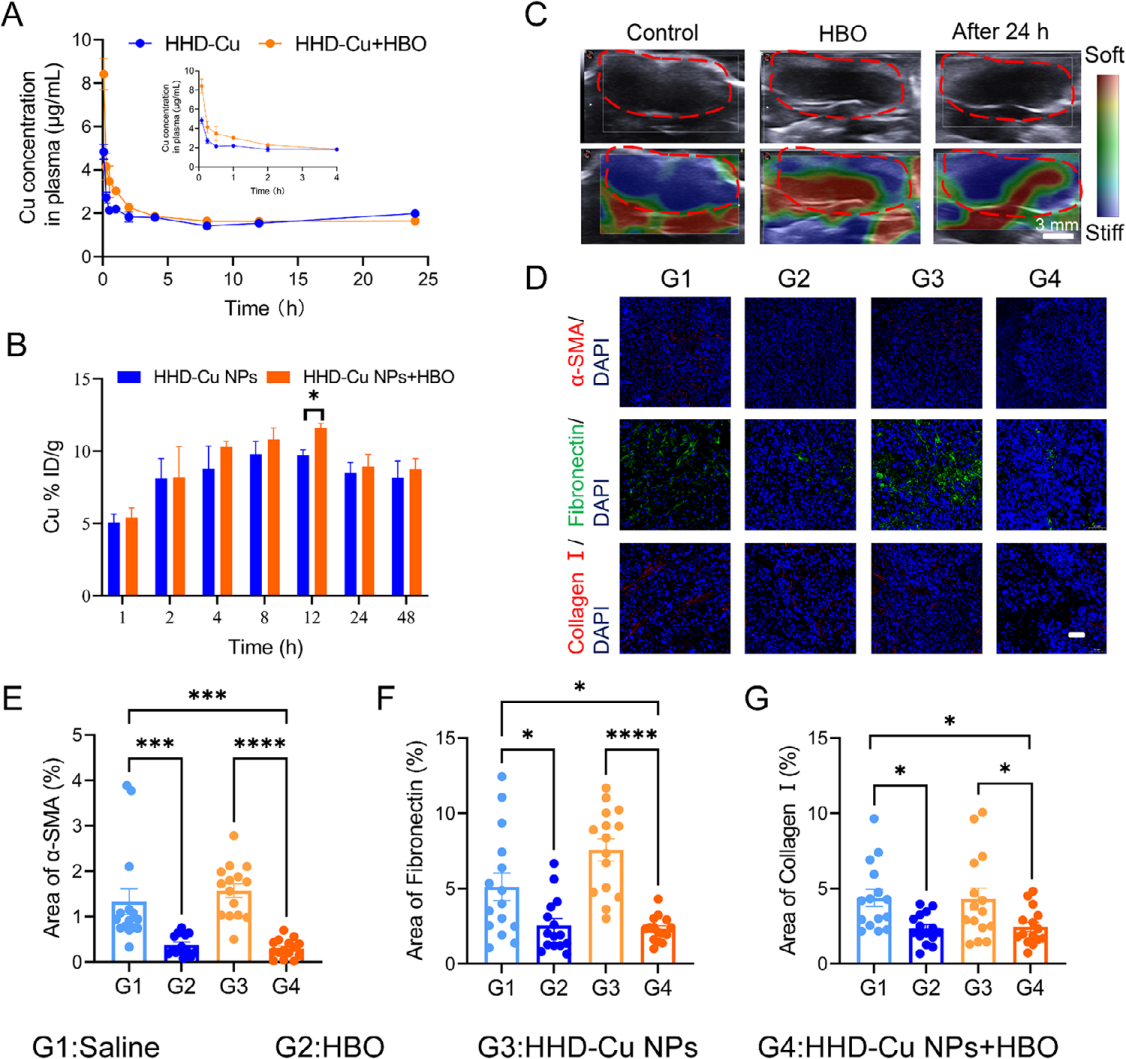
HBO promotes HHD‐Cu NPs tumor targeting delivery by depleting tumor ECM. (A) The blood circulation curve of intravenously injected HHD‐Cu NPs (*n* = 3 mice). (B) The tumor accumulation of Cu in tumor at different times post‐intravenous administrations of HHD‐Cu NPs. (C) Ultrasound elastography images of subcutaneous H22 tumors. (D) α‐SMA, Fibronectin and collagenIstaining of tumors. Percentage of the area of α‐SMA (E), fibronectin (F), and collagenI(G) (*n* = 15 independent replicates). Statistical significance was calculated by unpaired two‐sided Student's *t* test or one‐way ANOVA followed by post hoc Tukey's test and represented as the mean ± SEM. *p* values: *p < 0.05, **p < 0.01, ***p < 0.001, ****p < 0.0001, ns stands for not significant.

Prior to conducting antitumor efficacy experiments, we performed a series of in vivo experiments to determine how HBO affects tumor targeting delivery of HHD‐Cu NPs. Based on our previous finding that HBO facilitates tumor extracellular matrix (ECM) remodeling [32], we speculate that HBO could enhance HHD‐Cu NPs tumor accumulation. First, we divided BALB/c male mice into two groups of HHD‐Cu NPs and HHD‐Cu NPs + HBO, and then conducted pharmacokinetic experiments to monitor Cu in blood circulation after intravenous injection of HHD‐Cu NPs. Figure 6A and Table S1 show that HBO treatment significantly increases the half‐life time of HHD‐Cu NPs in plasma from 1.54 to 2.38 h and augments the area under curve (AUC) from 16.53 to 18.81 mg h L^−1^. Figure 6B and Figure S20 show the distribution of Cu in the tumor site and other organs, respectively. The results indicated that HBO enhanced the accumulation of HHD‐Cu NPs in tumor tissues. Notably, part of the accumulation in the liver completely disappeared within 48 h, while the accumulation in other organs was negligible. To further elucidate how HBO influences in vivo delivery behaviors of HHD‐Cu NPs. We used ultrasound imaging to detect changes in H22 subcutaneous tumors mechanical stiffness as well as blood flow velocity in HBO‐treated mice. Figure 6C and Figure S34 exhibit that the stiffness of subcutaneous tumors is significantly reduced after HBO treatment and remains relatively soft for 24 h. Accordingly, blood flow velocity is significantly augmented within H22 subcutaneous tumors (Figure S21), consistent with our previous report [31]. The abundant ECM of tumor tissues contributed to high mechanical stiffness, so we examined the changes in tumor ECM after various treatments. We divided mice into four groups including saline (G1), HBO (G2), HHD‐Cu NPs (G3), and HHD‐Cu NPs (G4), immunofluorescence staining for major components of tumor ECM including fibronectin, type I collagen, and α‐smooth actin (α‐SMA) in different groups are presented in Figure 6D–G, which reveal that HBO treatment significantly reduces tumor ECM, with decreases over 50% in all of these components.

In conclusion, HBO degrades tumor ECM, prolongs plasma circulation time of HHD‐Cu NPs, and enhances HHD‐Cu NPs tumor accumulation and enrichment. HBO has the potential to enhance the antitumor efficacy of HHD‐Cu NPs.

### 2.6 Antitumor Effect and Mechanism of HBO and HHD‐Cu NPs Combination Treatments

**FIGURE 7:**
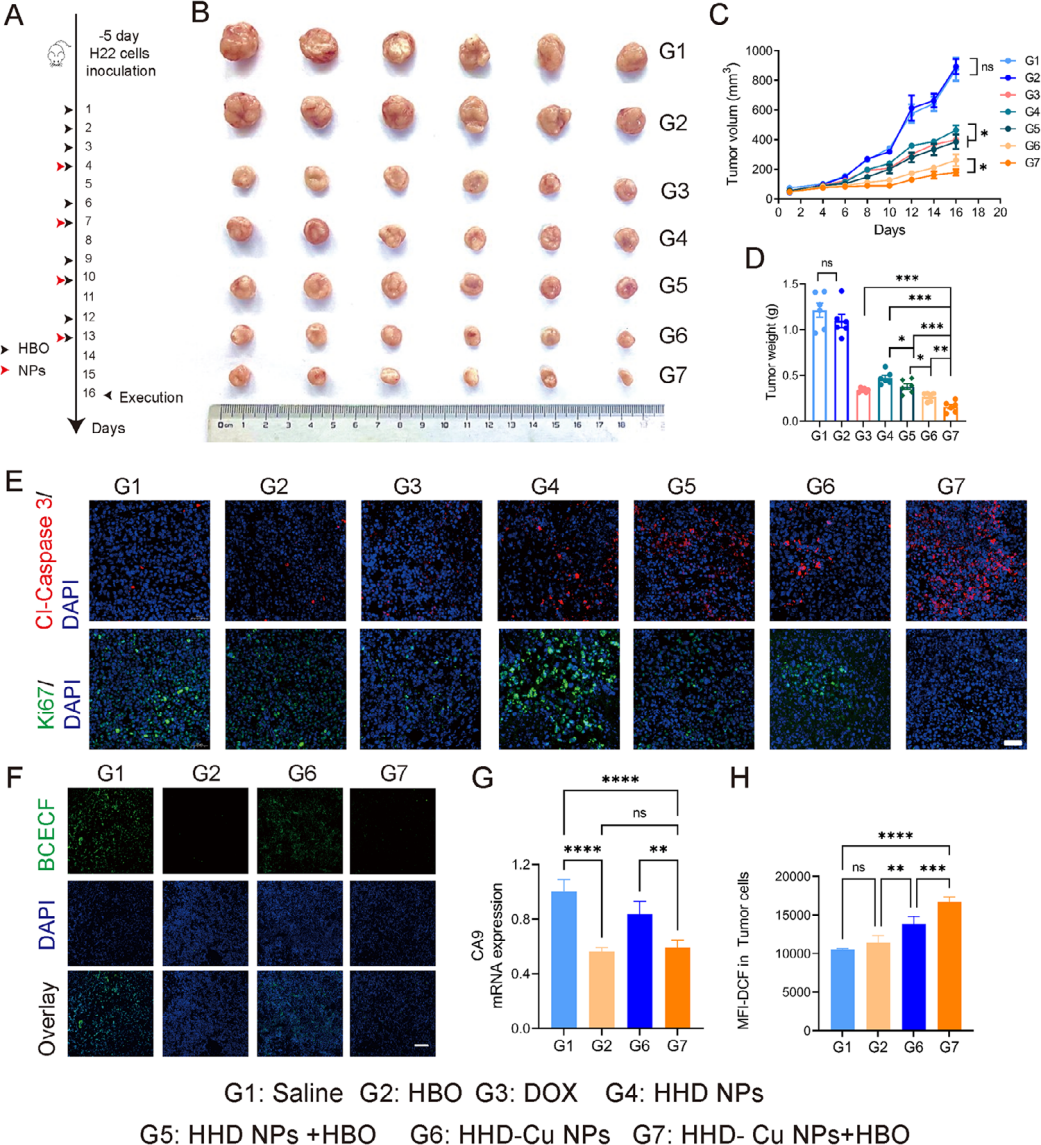
Antitumor effect and mechanism of HBO combined with HHD‐Cu NPs. (A) Schematic diagram of subcutaneous H22 tumor modeling and treatment protocol. (B) Photographic images of tumors excised from different groups after various treatments. (C) Tumor volumes and (D) tumor weight of the mice in various treatment groups (*n* = 6 mice). (E) Cleaved caspase‐3 and Ki‐67 staining images of tumor after different treatments (Scale bar: 50 µm). (F) BCECF staining images of tumor after different treatments (Scale bar: 100 µm). (G) CA9 mRNA as normalized to β‐actin of tumor cells after various treatments. (H) ROS levels in tumor cells after different treatments identified by flow cytometry. Statistical significance was calculated by unpaired two‐sided Student's *t* test or one‐way ANOVA followed by post hoc Tukey's test and represented as the mean ±SEM. *p* values: *p < 0.05, **p < 0.01, ***p < 0.001, ****p < 0.0001, ns stands for not significant.

Inspired by promising in vitro results, we established H22 subcutaneous tumor model to assess in vivo antitumor efficacy of HHD‐Cu NPs + HBO. When tumor volume reached 50 mm^3^, tumor‐bearing mice were randomized into groups including saline (G1), HBO (G2), DOX (G3), HHD NPs (G4), HHD NPs + HBO (G5), HHD‐Cu NPs (G6), and HHD‐Cu NPs + HBO (G7). Figure 7A shows our treatment strategy. According to Figure 7B–D, it was evident that the antitumor effects of HHD‐Cu NPs + HBO group (G7) and HHD NPs + HBO group (G5) were superior to those of HHD‐Cu NPs group (G6) and HHD NPs group (G4). HHD‐Cu NPs + HBO (G7) achieves the greatest tumor suppression, with a tumor inhibition rate reaching 87% (Figure S22). To assess the safety of the combination therapy, we monitored the body weights of mice during the treatment period. Figure S23 illustrates that, DOX group (G3) shows an evident decrease in the body weight of mice. However, there was no significant change in the body weights of mice in all other groups. In addition, no apparent pathological lesions are observed in H&E staining of major organs (Figure S24) and routine blood test (Figure S25), suggesting excellent biocompatibility of HHD‐Cu NPs + HBO. These results confirm that HBO could boost antitumor effects of HHD‐Cu NPs while triggering insignificant side effect.

To investigate the mechanism of HHD‐Cu NPs + HBO treatment, we conducted immunofluorescence staining and flow cytometry experiments on tumor tissues. First, tumor sections were stained for cleaved‐Caspase 3 to characterize apoptosis and Ki67 to assess proliferation activity, respectively. As illustrated in Figure 7E, compared to other groups, HHD‐Cu NPs + HBO group (G7) demonstrates a significant increase in apoptosis and a decrease in proliferation. Besides, HHD‐Cu NPs treated groups (G6 and G7) exhibit more inhibition of tumor proliferation and better induction of apoptosis than HHD NPs treated groups (G4 and G5). Meanwhile, flow cytometry of Annexin V/7‐AAD staining detected apoptosis (Figures S26 and S27) and illustrated a similar trend. BCECF‐AM staining further indicated a significant reduction in BCECF fluorescence intensity in HBO‐treated groups (G2 and G7) compared to non‐HBO treated groups (G1 and G6) (Figure 7F), suggesting a decrease in intracellular pH of tumor cells. To examine the underlying mechanism of pHi decrease, we also tested mRNA expressions of pH‐regulatory genes (CA9, MCT4, NHE1, NBC, and V‐ATPase) in tumor tissues (Figure 7G and Figure S28). HBO treated groups (G2 and G7) showed a downregulation of these genes, especially CA9, MCT4, NHE, compared to non‐HBO‐treated groups (G1 and G6). HHD‐Cu NPs + HBO (G7) treatment caused the most pronounced decrease among all groups. Subsequently, ROS levels were evaluated by DCFH‐DA staining of single‐cell suspension of tumor tissues. With flow cytometry analysis, Figure 7H illustrates that HHD‐Cu NPs (G6) increased ROS content within tumor cells. However, HHD‐Cu NPs + HBO (G7) further upsurged ROS level, showing a 59.3% increase compared to the saline group (G1).

Collectively, antitumor efficacy test in H22 subcutaneous tumor model confirmed that HBO suppressed pH‐regulatory genes such as CA9, regulated tumor pH, and augmented therapeutic effects of HHD‐Cu NPs. HHD‐Cu NPs + HBO significantly elevated intracellular ROS, induced apoptosis, and robustly eradicated tumor cells.

### 2.7 HBO Boosts HHD‐Cu NPs to Eliminate CSCs in H22 Subcutaneous Tumors

**FIGURE 8:**
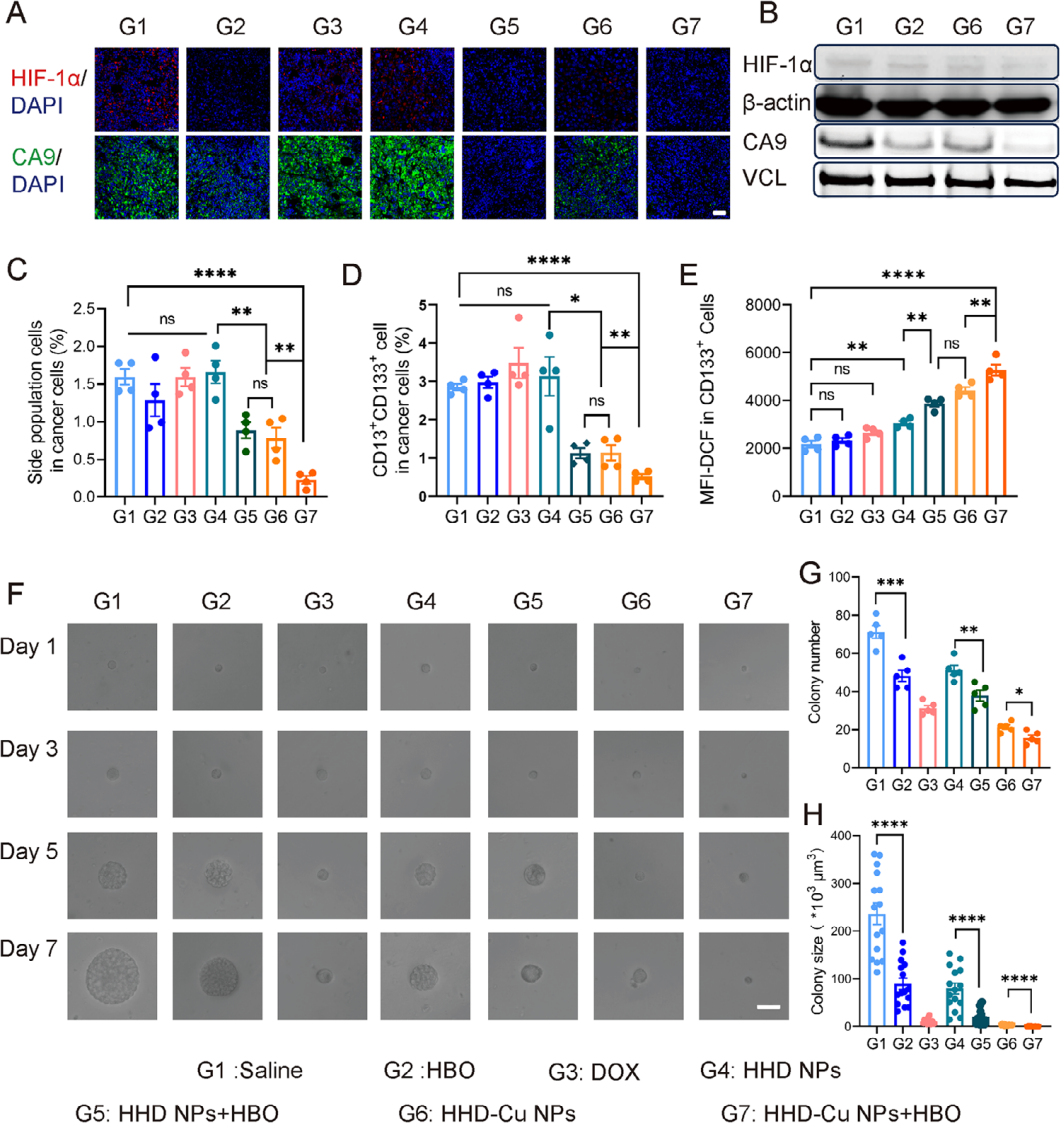
HBO boosts HHD‐Cu NPs to eliminate CSCs in H22 subcutaneous tumors. (A) HIF‐1α and CA9 staining of tumor tissues retrieved from the mice post various treatments (Scale bar: 50 µm). (B) Western Blot detection of HIF‐1ɑ and CA9 expression in tumors. Percentage of CSCs in tumor tissues after different treatments characterized by flow cytometry and identified with side population cells (C), CD13^+^CD133^+^ (D), and ROS level in CSCs (E); (*n* = 4 mice). (F) The 3D tumor spheres growth of tumor cells seeded in soft 3D fibrin (scale bar: 20 µm). (G) The colony number and (H) colony size at day 7. Statistical significance was calculated by unpaired two‐sided Student's *t*‐test or one‐way ANOVA followed by post hoc Tukey's test and represented as the mean ± SEM. *p* values: *p < 0.05, **p < 0.01, ***p < 0.001, ****p < 0.0001, ns stands for not significant.

Following the confirmation of the prominent therapeutic efficacy of HHD‐Cu NPs + HBO in H22 subcutaneous tumors, we sought to investigate whether our combination strategy exhibits suppression effects against CSCs. Figure 8A displays immunofluorescence staining for HIF‐1α and CA9 in tumor tissues from different treatments. The fluorescence intensity of HIF‐1α and CA9 significantly decreased in all HBO‐treated groups (G2, G5, and G7) compared to corresponding non‐HBO‐treated groups (G1, G4, and G6). Notably, the percentage of fluorescence area of HIF‐1α and CA9 in HHD‐Cu NPs + HBO group (G7) markedly decreased (Figure S29). According to western blot results in Figure 8B, HBO significantly reduces HIF‐1α and CA9 protein expressions in H22 subcutaneous tumors, consistent with immunofluorescence staining results. Given that HBO downregulated CA9, which overexpresses in CSCs, we examined the proportions of CSCs in H22 subcutaneous tumors. We conducted flow cytometry to measure the proportion of side population (SP) cells [50] in H22 subcutaneous tumor single‐cell suspension. Figure 8C and Figure S30 show that the proportion of SP cells significantly decreased in HBO‐treated groups (G5 and G7) compared with non‐HBO‐treated groups (G4 and G6). Additionally, HHD‐Cu NPs treated groups (G6 and G7) resulted in a notable reduction in the proportion of SP cells compared to HHD NPs groups (G4 and G5) and groups without nanoparticles (G1, G2, and G3). Crucially, the proportion of SP cells in the HHD‐Cu NPs + HBO group (G7) sharply decreased to 0.23% compared to that of 1.60% in the saline group (G1). Since surface marker CD13 plays a critical role in reducing ROS‐induced DNA damage [51], we chose CD13^+^CD133^+^ as a marker to detect the proportion of intratumoral CSCs. Figure 8D and Figure S31 show that HBO, DOX, or HHD NPs (G2, G3, and G4) did not decrease CSCs proportions, but HHD‐Cu NPs treated groups (G6 and G7) exhibited a significant reduction in CSCs proportion compared to all other groups. The most effective treatment, HHD‐Cu NPs + HBO (G7) reduced the proportion of CD13^+^CD133^+^ populations from 2.85% in the saline group (G1) to 0.52%. To assess the combination treatment's ability to induce ROS in CSCs, DCFH‐DA probe was used to measure ROS levels within CD133^+^ cancer cells. Figure 8E demonstrates that HBO‐treated groups (G5 and G7) exhibited higher ROS levels compared to the corresponding non‐HBO groups (G4 and G6). HHD‐Cu NPs treated groups (G6 and G7) displayed the most potent ROS induction capability, followed by HHD NPs treatment (G4 and G5), and free DOX (G3). Overall, HHD‐Cu NPs + HBO (G7) treatment exhibited the strongest induction of ROS, with a 142.7% increase compared to the saline group (G1).

After confirming that HBO decreases CSCs proportions in tumor tissues and induces ROS generation, we validated whether HHD‐Cu NPs + HBO inhibited the proliferation and self‐renewal of CSCs. Single‐cell suspension from H22 subcutaneous tumors of different treated mice was cultured within 3D fibrin gels in 96‐well plates for 7 days. The ability to form spheres is tested and presented in Figure 8F. With time, HBO‐treated groups (G2, G5, and G7) exhibited smaller tumor sphere diameters compared to corresponding non‐HBO‐treated groups (G1, G4, and G6). Tumor spheres in the HHD‐Cu NPs + HBO group (G7) showed almost no growth. On the 7th day, diameters of 15 representative tumor spheres per group are measured and plotted in Figure 8G, while Figure 8H demonstrates the number of tumor spheres per well. HBO treatment (G2, G5, and G7) significantly inhibited tumor spheres in number and size compared to corresponding non‐HBO treatments (G1, G4, and G6). Of note, HHD‐Cu NPs + HBO group (G7) exhibited fewer than 20 tumor spheres with an average volume of less than 700 µm^3^ on the 7th day. This observation aligns with the results of H22 cancer cells cultured within 3D fibrin gels (Figure S18). In conclusion, HBO, in combination with HHD‐Cu NPs, significantly inhibited both the stemness and sphere‐forming ability of tumor cells in H22 subcutaneous tumors.

## 3 Discussion

In this work, we found for the first time that HBO functions to regulate pH for both tumor cells and CSCs, which in turn enhances the antitumor efficacy of pH‐responsive HHD‐Cu NPs. The mechanism lies in that HBO alleviates hypoxia, induces a decrease in pHi and a rise in pHe, and improves tumor targeting delivery of HHD‐Cu NPs, DOX release, and efficiency of copper‐induced CDT. An acidic tumor microenvironment is associated with hypoxia [52, 53]; hypoxic solid tumors undergo metabolic alterations and upregulate acid efflux mechanisms mediated by CA9, among others, thereby leading to extracellular acidification [7]. These changes are pronounced in CSCs, as CA9 is overexpressed in CSCs [10] and correlates with poor prognosis [1]. The acidic tumor microenvironment has been shown to correlate with tumor progression, drug resistance, and metastasis [5, 8]. Therefore, regulating the acidic tumor environment has a positive effect on tumor therapy. On the one hand, oral administration of sodium bicarbonate (NaHCO_3_) or Tris‐base buffer elevated tumor extracellular pH, inhibited tumor metastasis, [54, 55] and enhanced immunotherapeutic efficacy [56]. On the other hand, inhibition of pH‐regulatory proteins boosted the antitumor efficacy of a variety of tumor therapies and significantly suppressed CSCs [10, 27, 57]. Since tumor pH regulation is associated with hypoxia and HBO was found to alleviate hypoxia in our previous studies [31, 32, 34], we wonder if HBO could inhibit pH‐regulatory proteins and verify this hypothesis in transcription level and with pH measurements. HBO inhibits not only CA9 but also a series of pH‐regulatory proteins, including MCT4, NHE1, NBC, and V‐ATPase (Figure 4 and Figure S28), thereby significantly inducing a decrease in pHi and an increase in pHe (Figures 4C,D and 5C; Figure S16). As such, HBO offers three advantages over other drugs that regulate tumor pH. First, HBO alleviates hypoxia upstream of pH regulation and shows a universal inhibitory effect on a wide range of pH‐regulated proteins. Second, HBO enhances the deep penetration and tumor enrichment of nanomedicines. Third, HBO is a clinically approved therapy. Therefore, combining HBO with pH‐responsive nanomedicines has significant potential for clinical applications.

CSCs are usually located in tumor centers and form a specific niche, consisting mainly of hypoxic environment, low pH, and abundant stroma cells and tumor ECM [10, 17]. While there have been many studies involving how hypoxia and abundant tumor ECM provide a growth environment for CSCs [17, 58], in recent years, acidic tumor pH has also been proven to be an important factor in promoting tumor stemness [59]. HBO might augment the efficacy of HHD‐Cu NPs on killing CSCs via three manners. First, HBO depletes ECM [31, 32, 34] and reduces tumor stiffness, enhancing the deep penetration and tumor enrichment of nanomedicines and paving the way for killing CSCs. Second, HBO regulates tumor pH and reduces the stemness and self‐renewal capability of CSCs. Third, CSCs are more sensitive to oxidative stress than bulk tumor cells [26], thus •OH produced by HHD‐Cu NPs can effectively kill CSCs [27]. In summary, our study provides a new therapeutic paradigm for the treatment of CSCs, whereby alteration of the CSC niche by HBO, along with nanomedicine‐mediated chemotherapy or CDT, can produce a significant inhibitory effect on CSCs.

Regarding the effects of HBO on tumor therapy, our results shed new insights. First, HBO can upregulate many pathways related to cellular energy metabolism (Figure 1), which may lead to an increase in cellular metabolites. Although the lactate pathway, which is key for the survival of cancer cells and CSCs via the Warburg effect [13, 60], did not show significant alterations, TCA cycle upregulation may lead to an increase in cellular CO_2_ production and thus promote intracellular acidification [61]. This might be another plausible explanation for HBO‐induced intracellular acidification. Second, acidified extracellular pH is closely associated with immunosuppression [62, 63], and regulating tumor pH might thus improve the antitumor efficacy of immunotherapy [64]. Our previous study illustrated that HBO augmented the efficacy of PD‐1 antibody‐mediated immunotherapy in H22 hepatocellular carcinoma tumor model [34]. Besides depleting tumor ECM, regulating tumor pH might be another important factor. We believe that HBO plays a key role in regulating tumor metabolism as well as immunity. However, the underlying mechanisms need to be investigated further.

Although we have discovered a novel pH‐regulatory approach through which HBO boosts the antitumor effects of HHD‐Cu NPs, limitations exist in the current study. First, HBO may lead to pleiotropic effects we have not studied. For instance, transcriptome findings revealed that HBO promotes glycolysis (Figure 2C), contrary to the result that CA9 inhibitor SLC‐0111 suppresses glycolysis [2]. There might be other mechanisms under HBO's pH‐regulatory effect, further exploration is essential before clinical applications. Second, HBO downregulates mRNA expression of pH‐regulatory proteins (Figure 2), but pathways involving pH regulation do not exist in the current KEGG database [65]. Through GSEA, we have identified several pathways, but the only pathway containing carbonic anhydrase family is nitrogen metabolism, usually associated with nitrogen fixation [66] rather than pH regulation. The carbonic anhydrase family, as widespread pH‐regulatory proteins in organisms [4], and the pathways behind their pH‐regulatory function need to be explored further.

## 4 Conclusions

In summary, we have demonstrated that HBO is a safe and effective method for regulating tumor pH. Together with our rational designed dual‐acid‐responsive HHD‐Cu NPs, we achieve efficient DOX release and copper‐mediated CDT within acidified tumor cells and CSCs. Additionally, HBO effectively remodels tumor extracellular matrix, enhancing HHD‐Cu NP tumor accumulation and paving the way for HHD‐Cu NPs to target deep‐seated CSCs. Therefore, the combination of HBO and HHD‐Cu NPs inhibits not only bulk tumor cells but also CSCs, achieving potent antitumor efficacy. This study explores a new function of clinical‐widely used HBO and establishes a novel combination therapy for treating CSCs abundant hypoxic solid tumors.

## 5 Materials and Methods

### 5.1 Materials

Hydroxyethyl starch (HES, 130 kDa) with hydroxyethyl molar substitution of 0.4 was a gift from Wuhan Huazhong University of Science and Technology Life Science and Technology Co. (Wuhan, China). Adriamycin hydrochloride was purchased from Beijing Huafenglianbo Technology Co., Ltd. (Beijing, China). 4‐Nitrophenyl chloroformate was purchased from Energy Chemical (Shanghai, China). CuCl_2_·2H_2_O, methylene blue (MB) and 5,5‐dimethyl‐1‐pyrroline *N*‐oxide (DMPO) were bought from Aladdin Reagent (Shanghai, China) Co., Ltd. PAGE Gel One‐Step Preparation Kit and Cell Lysis Buffer for Western Blot and Immunoprecipitation were obtained from Affinibody LifeScience Co. Ltd (Wuhan, China). BCECF‐AM and Hoechst 33342 were purchased from Beyotime Institute of Biotechnology (Shanghai, China). AFTSpin Tissue/Cell Fast RNA Extraction Kit for Animal, 2X Universal SYBR Green Fast qPCR Mix and ABScript III RT Master Mix for qPCR with gDNA Remover were purchased from ABclonal Technology Co., Ltd (Wuhan, China). Annexin V‐APC/7‐AAD apoptosis kit was purchased from Multisciences (LianKe) Biotech, Co., Ltd (Hangzhou, China). DCFH‐DA and Verapamil were obtained from Med Chem Express (China). FITC Rat Anti‐Mouse CD13 (R3‐242) was purchased from BD Pharmingen (China). APC‐anti‐mouse CD133 (141208) was obtained from Biolegend Inc. (San Diego, CA. USA).

### 5.2 Cell Lines and Animals

The murine hepatocellular carcinoma cell line H22 was obtained from Shanghai Institutes for Biological Sciences (Shanghai, China). H22 cells were cultured using 1640 medium containing 10% FBS and incubated at 37°C in a cell culture incubator containing 5% CO_2_. Induction of hypoxic cells: The cells were incubated in a hypoxic cell culture incubator (1% O_2_, 5% CO_2_, 37°C) for at least 48 h. BALB/c mice (male, 18–20 g) were purchased from Beijing Vital River Laboratory Animal Technology Co., Ltd. (Beijing, China). Mice were housed in an animal facility with constant environmental conditions (room temperature, 21 ± 1°C; relative humidity, 40%–70%; 12 h light‐dark cycle). All mice were allowed to eat and drink freely. All animal experiments were approved by the Animal Care and Use Committee of Tongji Medical College of Huazhong University of Science and Technology (Wuhan, China). The experimental protocol was approved by the Animal Ethics Committee of Tongji Medical College, Huazhong University of Science and Technology (Wuhan, China). Animal Ethics Approval Program No. is 2019S924.

### 5.3 CSC Culture

H22 CSCs were generated by culturing H22 cells in a three‐dimensional fibrin gel with an elastic stiffness of about 90 Pa [45, 48]. 2 mg mL^−1^ fibrinogen was mixed in equal volume with H22 cell suspension (4 × 10^4^ cells mL^−1^). The mixture (50 µL well^−1^) was loaded into a 96‐well plate containing 1 µL thrombin (0.1 U µL^−1^). After incubation at 37°C for 20 min, 200 µL of RPMI 1640 medium with 10% FBS was added to each well, and the 3D tumor spheres were cultured in a cell culture incubator for 3–5 days. CSC cells were obtained by digesting the 3D tumor spheres using collagenase and dispase.

### 5.4 HBO Protocol

HBO is performed in a hyperbaric chamber [30, 31, 32]. Briefly, hypoxic cells or mice were placed in a hyperbaric chamber. After ventilation with 100% oxygen, the absolute pressure is slowly increased to 2.5 atmospheric (ATM) in 15 min and maintained for 90 min. Then, the chamber is slowly depressurized to atmospheric pressure in 15 min, and the cells or mice are carefully removed.

### 5.5 RNA‐seq Protocol

First, H22 cells were cultured in a hypoxic cell culture incubator (1% O_2_, 5% CO_2_, 37°C) for at least 48 h. Then hypoxic H22 was divided into two groups: HBO and hypoxia (control). HBO group was treated as in the previous description. Each group (*n* = 4) was processed to extract a total of 5 million cells for the construction of mRNA libraries. A specific quantity of RNA samples was subjected to thermal denaturation to unfold secondary structures. Oligo(dT) magnetic beads were employed for mRNA enrichment, and the obtained mRNA underwent fragmentation through the addition of a disruption reagent at an appropriate temperature for a specified duration. A single‐stranded cDNA was synthesized by preparing a reaction system for one‐strand synthesis and implementing the corresponding reaction program. Subsequently, a double‐stranded cDNA was synthesized by preparing a reaction system for two‐strand synthesis and setting the reaction program accordingly. The double‐stranded cDNA ends were repaired in a reaction system, and an adenine (A) base was added to the 3′ end. An adapter ligation reaction system was prepared, and the reaction program facilitated the connection between the adapter and cDNA. A PCR reaction system was then prepared, and the reaction program was set to amplify the products. Following library quality control, PCR products were denatured into single strands, and a ligation reaction system was prepared with the corresponding program to obtain single‐stranded circular products. Linear DNA molecules that remained un‐circularized were digested, and the resulting single‐stranded circular DNA molecules underwent rolling‐circle replication to form DNA nanoballs (DNBs) containing multiple copies. The obtained DNBs were loaded onto a high‐density DNA nanochip, and sequencing was performed using combinatorial probe‐anchor synthesis (cPAS) technology. The raw sequencing data were filtered using SOAPnuke (v1.5.6) [67] to obtain clean data. Subsequently, HISAT2 was employed for reference genome alignment [68]. Data analysis, visualization, and exploration were conducted using R studio (R‐4.3.1). Differential gene expression analysis was performed using edgeR (v4.0.3) [37] with conditions set at *Q* value ≤ 0.05 or p.adjust ≤ 0.001. KEGG (https://www.kegg.jp/) enrichment analysis, GO (http://www.geneontology.org/) enrichment analysis, and GSEA enrichment analysis, along with relevant graphical representations, were carried out using clusterProfiler (v4.10.0) [38]. The expression clustering heatmap for genes across different samples was generated using ComplexHeatmap (v2.18.0) [69].

### 5.6 TCGA Analysis

Initially, data retrieval was conducted using easyTCGA (v0.0.1.8000) to download transcriptomic profiles and clinical follow‐up information for 377 patient samples from the TCGA‐LIHC database. Patient data with TPM = 0 for CA9 and HIF‐1α, as well as CD133 (PROM1), were excluded when plotting the correlation. The ggstatsplot package (v0.12.1) was utilized to generate correlation plots, and Pearson correlation coefficients along with *p*‐values were analyzed. Patient‐specific CA9 expression labels were established, with values above the median considered as high CA9 expression, and values below as low CA9 expression. The survival package (v3.5‐5) was employed to create Kaplan‐Meier survival curves for patients based on CA9 expression levels, and *p*‐values were calculated.

### 5.7 Synthesis of pH‐Responsive HES‐Hyd‐DOX Conjugate

The synthesis steps of HES‐NPC are as follows [42].

1. Dissolve 1 g of HES in a reaction vial containing 24 mL of ultrapure water and vigorously stir in an ice bath.
2. Add 1 mL of 80 mg mL^−1^ NaOH solution to the reaction vial.
3. Dissolve 4‐nitrophenyl chloroformate in 10 mL of dichloromethane and slowly add it dropwise to the reaction vial. Allow the reaction to proceed at room temperature for approximately 1–2 h until the color of the reaction mixture changes from yellow to white.
4. Stop the reaction, add 200 mL of isopropanol, centrifuge, discard the supernatant, and precipitate HES‐NPC.

The synthesis steps of HES‐NH‐NH_2_ are as follows.

1. Weigh 500 mg of HES‐NPC into a reaction vial and add 5 mL of dimethyl sulfoxide to dissolve it completely.
2. Slowly add 500 µL of hydrazine hydrate to the reaction mixture and stir for 3 h at room temperature.
3. Gradually, drip the reaction mixture into 200 mL of isopropanol to precipitate the product. Centrifuge the mixture and wash the precipitate three times with petroleum ether to obtain the target product, HES‐NH‐NH_2_.
4. Vacuum dry the product for storage and further use.

The synthesis steps of HES‐hyd‐DOX are as follows.

1. Weigh 200 mg of HES‐NH‐NH_2_ into a reaction vial and add 3 mL of dimethylformamide to dissolve it completely.
2. Sequentially add 30 µL of triethylamine, 20 µL of acetic acid, and a dimethylformamide solution of DOX‐HCl (40 mg of DOX dissolved in 1 mL of DMF, with an additional 10 µL of triethylamine) to the reaction mixture.
3. Stir the reaction mixture at room temperature under N_2_ protection for 48 h.
4. Dialyze the reaction mixture using ultrapure water for 3 days.
5. Obtain the target product, HES‐hyd‐DOX.
6. Further dialyze HES‐hyd‐DOX for 3 days, then lyophilize and store it under vacuum drying.
7. The grafting rate of DOX is 8.5%.

The structure of HES‐hyd‐DOX was characterized using nuclear magnetic resonance hydrogen spectroscopy (^1^H NMR), with deuterated dimethyl sulfoxide (DMSO‐d6) as a solvent and HES‐hyd‐DOX concentration of 100 mg mL^−1^, on a nuclear magnetic resonance spectrometer (AscendTM 600 MHz, Bruker).

### 5.8 Preparation of HHD‐Cu NPs

HES‐hyd‐DOX containing 8.5 mg of DOX was dissolved in 9 mL of ultrapure water. Then, 0.2 mL of dichloromethane was added, and the mixture was sonicated at 150 W for 2 min. The dichloromethane was removed by rotary evaporation, resulting in an aqueous solution of HHD NPs. Subsequently, a 27 mg mL^−1^ CuCl_2_ solution was slowly added dropwise to the HHD NPs and stirred at room temperature overnight. During this process, the color of the solution changed from orange‐red to purple. The HHD‐Cu NPs were obtained through subsequent purification using dialysis. Finally, the HHD‐Cu NPs were lyophilized, dried, and stored away from light.

### 5.9 Characterization of HHD‐Cu NPs

Hydrodynamic diameter distribution as well as zeta potential of HHD‐Cu NPs were examined using a dynamic light scattering (DLS, Malvern, Zetasizer Nano‐ZS, UK), and data were collected using Zetasizer software (version 7.13). The particle size and morphology of HHD‐Cu NPs were observed using a transmission electron microscope (TEM, HT7700, HITACHI Co., Japan). In detail, 10 µL of HHD‐Cu NPs solution was added dropwise to the surface of 300 mesh carbon‐coated copper mesh, and the liquid on the surface was left for 24 h to evaporate fully. Then, HHD‐Cu NPs were characterized on a TEM. The absorption spectra of the samples were measured using a UV spectrophotometer (Lambda 35, PerkinElmer Instruments Co., Ltd., Shanghai, China), while the fluorescence intensity of the samples was detected using a fluorescence spectrophotometer. Elemental distribution and valence states in HHD‐Cu NPs were examined using field emission transmission electron microscopy (FTEM, FEI Technai G2F30) and an X‐ray photoelectron spectrometer (XPS, AXIS‐ULTRA DLD‐600 W). The generation of free radicals in HHD‐Cu NPs was probed using electron spin resonance spectroscopy (ESR, A300, Bruker Co., Germany). The content of Cu in HHD‐Cu NPs was quantified by the nitrolysis method. Typically, a certain amount of HHD‐Cu was weighed and placed in a nitrolysis bottle, concentrated nitric acid and perchloric acid were added, and the nitrolysis process was heated up to 300°C. Then, the concentration of Cu in the nitrolysis solution was measured by an inductively coupled plasma optical emission spectrometer (ICP‐OES, PerkinElmer Ltd., Co., USA).

### 5.10 HHD‐Cu NPs Stability Test

The PBS solution containing HHD‐Cu NPs was placed on a shaker at 37°C for 7 days, and the stability was assessed by measuring the change in particle size using DLS every day.

### 5.11 HHD‐Cu NPs pH‐Responsive Test

HHD‐Cu NPs were dissolved in phosphate buffer (0.01 M) at pH 7.4, 6.5, and 5, respectively, and then placed on a shaker at 37°C for 12 h. Changes in particle size of HHD‐Cu NPs were monitored using DLS, while changes in fluorescence intensity were measured using a fluorescence spectrophotometer.

### 5.12 Release of DOX and Cu^2+^ Under Different pH Conditions

DOX release experiments were conducted using the dialysis method. In brief, 1 mL of HHD‐Cu NPs solution (DOX concentration of 300 µg mL^−1^) was placed in a 3500 Da dialysis bag. These bags were then submerged in different pH phosphate buffers (30 mL, pH 7.4, pH 6.5, pH 5, 0.5% V/V Tween‐80) and incubated at 37°C on a shaker with gentle agitation. Subsequently, 200 µL of release solution was withdrawn at various time points (0, 1, 2, 4, 8, 12, 24, and 48 h) and replaced with an equal volume of fresh buffer. The concentration of DOX in the release solution was determined using a microplate reader (Molecular Devices, Flex Station 3, USA), with an excitation wavelength of 488 nm and an emission wavelength of 565 nm. Each set of experiments was conducted in triplicate.

Cu^2+^ release experiments were carried out using the same method, with two differences being that the release solution was Tween‐80 free and the volume of release solution removed was 5 mL, which was then replenished by adding an equal volume of fresh buffer. The concentration of Cu^2+^ in the release solution was detected using ICP‐OES. Each set of experiments was conducted in triplicate.

### 5.13 Chemodynamical Activity of HHD‐Cu NPs

Hydroxyl radical production from H_2_O_2_ catalyzed by HHD‐Cu NPs under different pH conditions was examined using a methylene blue colorimetric method [42]. Specifically, 1.5 mL of different pH buffers (pH 7.4, pH 6.5, and pH 5) were used to dissolve HHD‐Cu NPs (the concentration of Cu was 0.5 mM), which were mixed with an equal volume of GSH (1 mM), followed by addition of 3 µL of 30% hydrogen peroxide and MB solution (30 µL and 1 mg mL^−1^). Then, the reaction was carried out for 4 h in a shaker at 37°C. The absorption spectrum of MB was scanned using a UV spectrophotometer, and the decrease of UV absorption at 660 nm was monitored.

In addition, ·OH was detected by ESR spectroscopy using DMPO as a radical trapping agent [42]. HHD‐Cu NPs dissolved in different pH buffers were mixed with GSH and incubated for 2 h. Then, 10 mM DMPO, as well as hydrogen peroxide, was added to the reaction system, and hydroxyl radical detection was performed using ESR (A300, Bruker Co., Germany).

### 5.14 mRNA Transcription Level

Quantitative real‐time PCR (qRT‐PCR) was used to quantify RNA expression levels of CA9, MCT4, NBC, NHE1, V‐ATPase in H22 cells and tumor tissues. RNA of H22 cells or tumor tissues were isolated with AFTSpin Tissue/Cell Fast RNA Extraction Kit for Animal. RNA was reverse‐transcribed to cDNA following the protocol of ABScript III RT Master Mix for qPCR with gDNA Remover. qPCR reactions were performed using the Universal SYBR Green Fast qPCR Mix kit. All results use β‐actin as a normalized control.

The primer sequences are as shown below.

**Table:** 

Gene		Sequence
CA9 (Car9)	F primer	TGCTCCAAGTGTCTGCTCAG
	R primer	CAGGTGCATCCTCTTCACTGG
MCT4(Slc16a3)	F primer	TCACGGGTTTCTCCTACGC
	R primer	GCCAAAGCGGTTCACACAC
NBC (Slc4a7)	F primer	GCTGGCTACCACCTTGACTTG
	R primer	ACTTATAGACAACACTGTCGCAG
NHE (Slc9a1)	F primer	TCATCCACCTCGGATCTTCCC
	R primer	TCCTGAGAACAGGTAGCAGTC
ATPase (Atp6v1g2)	F primer	GAGGAGGCTCAAATGGAGGTG
	R primer	CTGAACCTGCCGTCTTGTG
β‐actin	F primer	GGCTGTATTCCCCTCCATCG
	R primer	CCAGTTGGTAACAATGCCATGT

### 5.15 Western Blot Analysis

H22 cells as well as tumor tissues were lysed in an ice bath using Cell Lysis Buffer for Western Blot and Immunoprecipitation for 15 min, followed by centrifugation at 14,000 g for 5 min to collect the supernatant. The protein concentration was measured using the BCA Protein Concentration Assay Kit. Add loading buffer to the mix and heat at 100°C for 10 min. Equal amounts of proteins (40 µg) were processed by SDS‐PAGE (10%) and transferred to PVDF (Millipore) membranes. After incubation with 5% skimmed milk for 1 h, membranes were incubated overnight at 4°C with primary antibodies diluted with Antibody Diluent [HIF‐1α (1:1000), CA9 (1:1000), β‐actin (1:5000), Vinculin (VCL) (1:2000)], followed by incubation with HRP‐conjugated mouse or rabbit secondary antibodies (1:5000). Signal intensity was detected using a Chemiluminescence Kit (Thermo Fisher Scientific, IL) on ChemiDoc XRS+ System (Bio‐Rad Laboratories, Inc).

### 5.16 The Detection of Intracellular and Extracellular pH

Methods for the detection of pHi in H22 cells. H22 cells were inoculated into 96‐well plates (5000 cells well^−1^), and incubation was continued under hypoxic (1% O_2_) conditions for 72 h. Cells in the HBO group were treated with HBO every 24 h. Methods for the detection of pHi in HUVEC cells were basically the same as H22 cells except that the control group was incubated at normoxic condition (21% O_2_). The measurement of pHi was calculated from fluorescence intensity of BCECF‐AM fluorescent probe. Briefly, BCECF‐AM fluorescent probe was prepared using HHBS buffer Hank's Balanced Salt Solution with 20 mM HEPES, without sodium bicarbonate. The cell culture medium was removed, the cells were washed three times using HHBS, and the cells were incubated with HHBS buffer containing BCECF‐AM probe for 30 min at 37°C, protected from light. Ratio testing is then performed at *λ*_ex_ = 440 nm; *λ*_em_ = 535 nm and *λ*_ex_ = 490 nm; *λ*_em_ = 535 nm using a FlexStation3 microplate reader (molecular devices). Calibration standard curves were prepared using the nigericin method as described in previous literature reports [2].

Methods for pHi detection of CSCs. The three‐day cultured 3D tumor spheres were divided into four groups and transferred to hypoxic conditions for an additional 72 h. The groups included a hypoxia group, an HBO group, an HHD‐Cu NPs group, and an HHD‐Cu NPs + HBO group. The HBO group and HHD‐Cu NPs + HBO group underwent HBO treatment every 24 h. After the second HBO treatment, the culture medium for the HHD‐Cu group and HHD‐Cu + HBO group was replaced with fresh medium containing HHD‐Cu NPs (DOX concentration of 1 µg mL^−1^) for continued hypoxic incubation for 24 h. Subsequently, another round of HBO treatment was administered. 3D tumor spheres were then enzymatically digested into single‐cell suspension, and BCECF‐AM staining was performed on the dissociated H22 CSCs. The method of monitoring CSCs pHi was the same as described in H22 cells pHi detection.

Change in extracellular pH (pHe) is measured using a pH meter. H22 cells were inoculated into 6‐well plates (4 × 10^5^ cells well^−1^), and incubation was continued under hypoxic (1% O_2_) conditions for 48 h. Specifically, the pH of the cell culture medium under hypoxic conditions is first measured. Subsequently, after HBO treatment, the cells are further cultured in the hypoxic incubator, and the pH of the culture medium is measured again after 24 h. The difference between the pH value obtained from the latter measurement and that obtained from the former measurement represents the change in extracellular pH.

### 5.17 Cell Viability of H22 Cells

H22 cells in hypoxic culture were collected. Then, H22 cells were inoculated into 96‐well plates (5000 cells well^−1^), and incubation was continued under hypoxic (1% O_2_) conditions H22 cells were then incubated with HHD‐Cu NPs and cultured for another 48 h under hypoxia (1% O_2_) or hypoxia plus HBO (two times). Cell viability was measured using the Cell Counting Kit ‐8 (CCK‐8 kit).

### 5.18 Cell Viability of H22 CSCs

The 3‐day cultured 3D tumor spheres were transferred to a hypoxic environment for an additional 48 h. Subsequently, they were enzymatically digested into a single‐cell suspension of H22 CSCs. The cells were then cultured for an additional 12 h with a medium containing HHD‐Cu NPs. Two experimental groups were established based on different treatments: hypoxia group and HBO group. The HBO group underwent HBO treatment every 24 h, totaling two sessions. Cell viability was assessed using CCK‐8 kit.

### 5.19 Cell Viability of HUVEC Cells

HUVEC cells in DMEM culture were collected. Then, HUVEC cells were inoculated into 96‐well plates (5000 cells well^−1^) and incubation was continued under normoxia (21% O_2_) condition. HUVEC cells were then incubated with HHD‐Cu NPs and cultured for another 48 h. Cell viability was measured using CCK‐8 kit.

### 5.20 Apoptosis and Necrosis Assay in Vitro

H22 cells, cultivated under hypoxic conditions, were seeded into a 6‐well plate (4 × 10^5^ cells well^−1^) and incubated overnight. The cells were then divided into six groups: hypoxia (G1), HBO (G2), HHD NPs (G3), HHD NPs + HBO (G4), HHD‐Cu NPs (G5), and HHD‐Cu NPs + HBO (G6). Subsequently, cells were treated with HHD NPs or HHD‐Cu NPs (DOX concentration: 1 µg mL^−1^) for 12 h. Afterward, the cells underwent hypoxic treatment and hypoxic treatment combined with HBO for an additional 12 h. Cell apoptosis was analyzed using Annexin V‐APC/7‐AAD staining by flow cytometry. The gating strategy is shown in Figures S13 and S28.

### 5.21 Intracellular ROS Detection

DCFH‐DA (MCE, Shanghai, China) was utilized to measure intracellular ROS levels. Specifically, H22 cells cultured under hypoxic conditions were seeded into a 6‐well plate (4 × 10^5^ cells well^−1^) using a serum‐free medium. The cells were categorized into six groups: hypoxia (G1), HBO (G2), HHD NPs (G3), HHD NPs + HBO (G4), HHD‐Cu NPs (G5), and HHD‐Cu NPs + HBO (G6). After treating the cells with HHD NPs and HHD‐Cu NPs (DOX concentration: 1 µg mL^−1^) for 4 h, DCFH‐DA (10 µM) was added for cell staining. Concurrently, HBO treatment was performed. The cells were washed with PBS, and intracellular ROS levels after different treatments were quantified using flow cytometry.

H22 CSCs intracellular ROS levels were also assessed using DCFH‐DA. Specifically, 3D tumor spheres cultivated for three days were transferred to a hypoxic environment for an additional 48 h. The groups were the same as previously described. After drug treatment (DOX concentration: 1 µg mL^−1^) for 4 h, DCFH‐DA was added to stain 3D tumor spheres, and HBO treatment was simultaneously administered. After washing the cells with PBS, the ROS levels within 3D tumor spheres were characterized using confocal laser scanning microscopy (CLSM, OLYMPUS FV3000). Additionally, 3D tumor spheres were digested into single‐cell suspensions, and flow cytometry was employed to quantify intracellular ROS levels post different treatments. The HUVEC cell lines were treated similarly to the above, but only they were divided into two groups, normoxia and HBO.

### 5.22 HHD‐Cu NPs Pharmacokinetics and Biodistribution

For pharmacokinetic studies, H22 subcutaneous tumor‐bearing mice (*n* = 3) were intravenously injected with a 200 µL solution of HHD‐Cu NPs (Cu dose of 2 mg kg^−1^). Subsequently, blood samples (50 µL) were collected from the submandibular vein at predetermined time points (0.083, 0.25, 0.5, 1, 2, 4, 8, 12, and 24 h). The samples were subjected to overnight digestion with a mixture of nitric acid and perchloric acid (*V*/*V* = 4:1), followed by dilution, and Cu concentration was measured using ICP‐OES.

For biodistribution studies, H22 subcutaneous tumor‐bearing mice (*n* = 3) were intravenously injected with a 200 µL solution of HHD‐Cu NPs (Cu dose of 2 mg kg^−1^). Mice were euthanized at predetermined time points after administration (1, 2, 4, 8, 12, 24, and 48 h). Major organs (heart, liver, spleen, lung, and kidney) and tumor tissues were collected and digested with a mixture of nitric acid and perchloric acid (*V*/*V* = 4:1) at 300°C. After dilution, Cu concentration was measured using ICP‐OES. Further investigation into the impact of HBO on biodistribution was conducted. H22 subcutaneous tumor‐bearing mice (*n* = 3), pre‐treated with HBO three times, were intravenously injected with HHD‐Cu NPs (Cu dose of 2 mg kg^−1^). At predetermined time points after administration (1, 2, 4, 8, 12, 24, and 48 h), mice were euthanized, and tumors were collected, weighed, and digested. Cu concentrations in tumors were determined using ICP‐OES.

### 5.23 Tumor Tissue Stiffness Assessment

In mice bearing H22 subcutaneous tumors with a tumor volume of 300 mm^3^, the elasticity of tumor tissues was measured using the BaiSheng Elasticity Imaging Ultrasound Diagnostic Instrument (X8 Series) to assess changes in modulus following HBO treatment. Initially, elastic ultrasound imaging was performed on tumor tissues, followed by immediate HBO treatment. Subsequently, another ultrasound imaging session was conducted 24 h after the completion of HBO treatment.

### 5.24 In Vivo Pharmacodynamics Experiment

Male BALB/c mice aged 4 to 6 weeks were subcutaneously inoculated with H22 tumor cells at a density of 1 × 10^6^ cells per mouse. When tumor volume reached approximately 60 mm^3^, mice were randomly divided into seven groups (*n* = 6) for different treatments: Saline (G1), HBO (G2), DOX (G3), HHD NPs (G4), HHD NPs + HBO (G5), HHD‐Cu NPs (G6), and HHD‐Cu NPs + HBO (G7).

The HBO group mice underwent continuous HBO treatment for 3 days, once a day. On the 4th day, 7th day, 10th day, and 13th day, different drugs were intravenously injected through the tail vein, with a DOX dose of 4 mg kg^−1^. HBO treatment was performed one day before each administration and within 2 h after administration. Meanwhile, body weight and tumor volume were recorded every 3 days. Tumor volume (*V*) was calculated using the formula *V* = (tumor width^2^ × tumor length)/2. After completion of treatment, euthanasia was performed on the mice, and tumors were collected, photographed, and weighed.

### 5.25 Tumor Tissue CSCs Assessment

After completion of treatment, euthanasia was performed on mice (*n* = 4), and tumors were collected to assess CSCs in tumor tissues. First, tumors were cut into small pieces as much as possible and incubated with Collagenase IV (Biosharp) and DNAse I (Biosharp) at 37°C for 1 h. After digestion, tumor tissues were filtered through a 70 µm cell strainer to obtain single‐cell suspensions. Subsequently, cells were washed twice with PBS, resuspended in 300 µL PBS, and stained with specific antibodies. The identification of CSCs in tumor tissues was conducted using CD13^+^CD133^+^ cell analysis (Figure S25). For side population (SP) cell analysis, cells were incubated with 5 µg mL^−1^ Hoechst 33342 in combination with 200 or 400 µM Verapamil at 37°C for 90 min. After staining, flow cytometry was employed to analyze the samples [50, 70] (Figure S26).

### 5.26 Immunofluorescence Staining

Immunofluorescence staining was performed on tumor slices using an anti‐HIF‐1α fluorescent‐labeled antibody (PTG: 20960‐1‐AP) and CA9 fluorescent‐labeled antibody (PTG: 11071‐1‐AP). Additionally, to evaluate the impact of HBO on the extracellular matrix of tumor tissues, immunofluorescence staining was conducted on tumor slices using antibodies against fibronectin (Abcam: ab268020), collagen I (Boster: BA0325), and α‐SMA (Affinity, AF 1032). The apoptotic and proliferative status of tumor tissue was examined using immunofluorescence staining with Cleaved‐Caspase 3 (CST, Asp 175) and Ki67 (CST, 9129). Immunofluorescence staining of tumor slices was observed using CLSM.

### 5.27 Safety Evaluation

The body weight of the mice was monitored every 3 days throughout the treatment period. At the end of treatment, the mice were euthanized, and their major organs and tumor tissues were harvested for hematoxylin and eosin (H&E) staining. Additionally, blood samples were collected for blood biochemistry and routine blood tests to assess systemic side effects.

### 5.28 Statistical Analysis

Most measured data were displayed directly and normalized data were described as relative value. Data were presented as mean values ± SD or mean values ± SEM. The sample size for each statistical analysis was no less than 3. Statistical significance was calculated by unpaired two‐sided Student's *t*‐test or one‐way ANOVA. *P*‐values of <0.05 were considered statistically significant. Statistical analysis was performed using GraphPad Prism 8.0 software.

## Author Contributions

Z.F.L. designed and supervised the project. Z.F.L. and X.L.Y. acquired financial support. Q.Y.D., A.H., S.Y.L. Z.J.Z., X.C., Q.W., and X.W. performed experiments. Q.Y.D., A.H., and Z.F.L. analyzed and interpreted the data. Q.Y.D., A.H., and Z.F.L. wrote and revised the manuscript.

## Conflicts of Interest

The authors declare no conflicts of interest.

## Supporting information



Supporting Information

## Data Availability

The data that support the findings of this study are available from the corresponding author upon reasonable request.
