# Supplementary material for: Hyperbaric Oxygen Regulates Tumor pH to Boost Copper‐Doped Hydroxyethyl Starch Conjugate Nanoparticles Against Cancer Stem Cells
Source: Exploration (Beijing). 2025 Apr 3;5(4):e20240080. doi: 10.1002/EXP.20240080 (PMC12380064; doi:10.1002/EXP.20240080)
Supplement: Supplementary file 1 — Supporting Information [file EXP2-5-e20240080-s001.pdf]

Supporting Information for

Hyperbaric oxygen regulates tumor pH to boost copper-doped hydroxyethyl starch conjugate nanoparticles against cancer stem cells

Qingyuan Deng[a, #], Ao Hua[a, #], Shiyou Li[a], Zhijie Zhang[a], Xiang Chen[a], Qiang Wang[a], Xing Wang[a], Zhiqin Chu[f, g] Xiangliang Yang[a, b, c, d, e, *], Zifu Li[a, b, c, d, e, *]

[a] Department of Nanomedicine and Biopharmaceuticals, College of Life Science and Technology, Huazhong University of Science and Technology, Wuhan, 430074, P. R. China

[b] National Engineering Research Center for Nanomedicine, Huazhong University of Science and Technology, Wuhan, 430074, P. R. China

[c] Key Laboratory of Molecular Biophysics of Ministry of Education, Huazhong University of Science and Technology, Wuhan, 430074, P. R. China

[d] Hubei Key Laboratory of Bioinorganic Chemistry and Materia Medical, Huazhong University of Science and Technology, Wuhan, 430074, P. R. China

[e] Hubei Engineering Research Center for Biomaterials and Medical Protective Materials, Huazhong University of Science and Technology, Wuhan, 430074, P. R. China

[f] Department of Electrical and Electronic Engineering, The University of Hong Kong, Pokfulam Road, Hong Kong, P. R. China

[g] School of Biomedical Sciences, The University of Hong Kong, Hong Kong, P. R. China

[#] These authors contribute equally.

* Address correspondence to:

Zifu Li, Ph.D., Professor

Huazhong University of Science and Technology

1037 Luoyu Road, Wuhan, 430074, P. R. China

Email: zifuli@hust.edu.cn

Xiangliang Yang, Ph.D., Professor

Huazhong University of Science and Technology

1037 Luoyu Road, Wuhan, 430074, P. R. China

Email: yangxl@hust.edu.cn

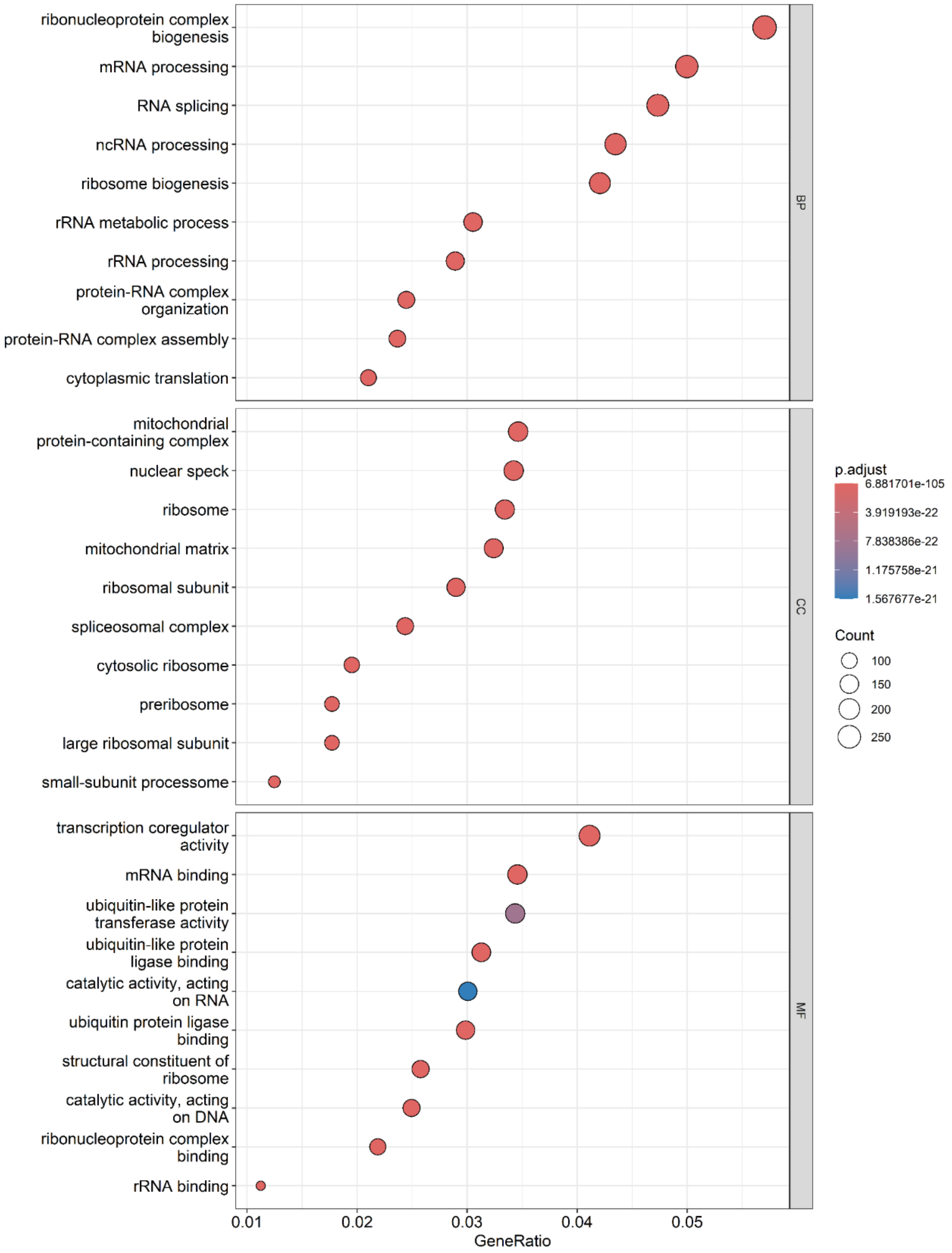

Figure S1 GO enrichment analysis of HBO compared to hypoxic group in transcriptome of H22 cells. The top ten differentially expressed pathways in biological processes (BP), cellular components (CC), and molecular functions (MF), were presented.

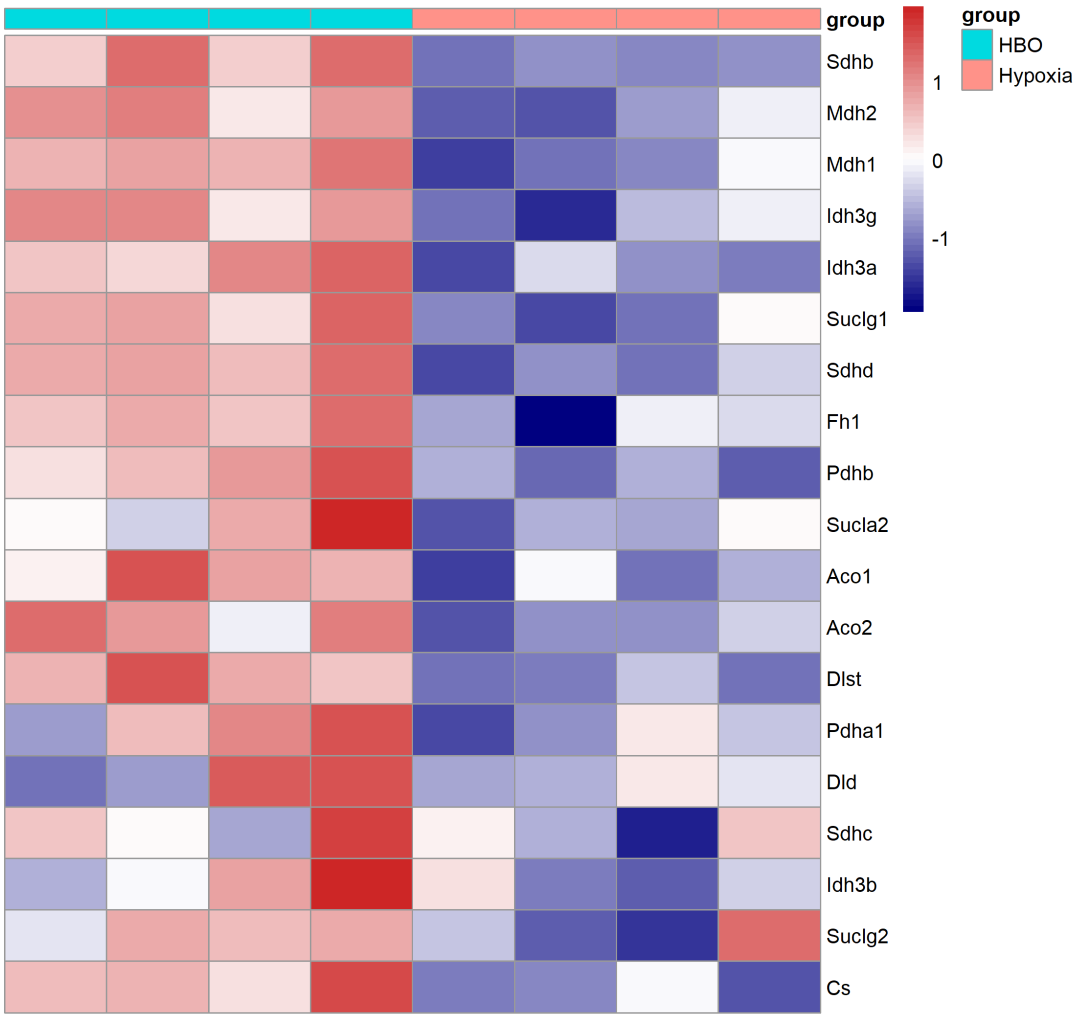

Figure S2 Heatmap of genes that were significantly altered in the tricarboxylic acid (TCA) cycle.

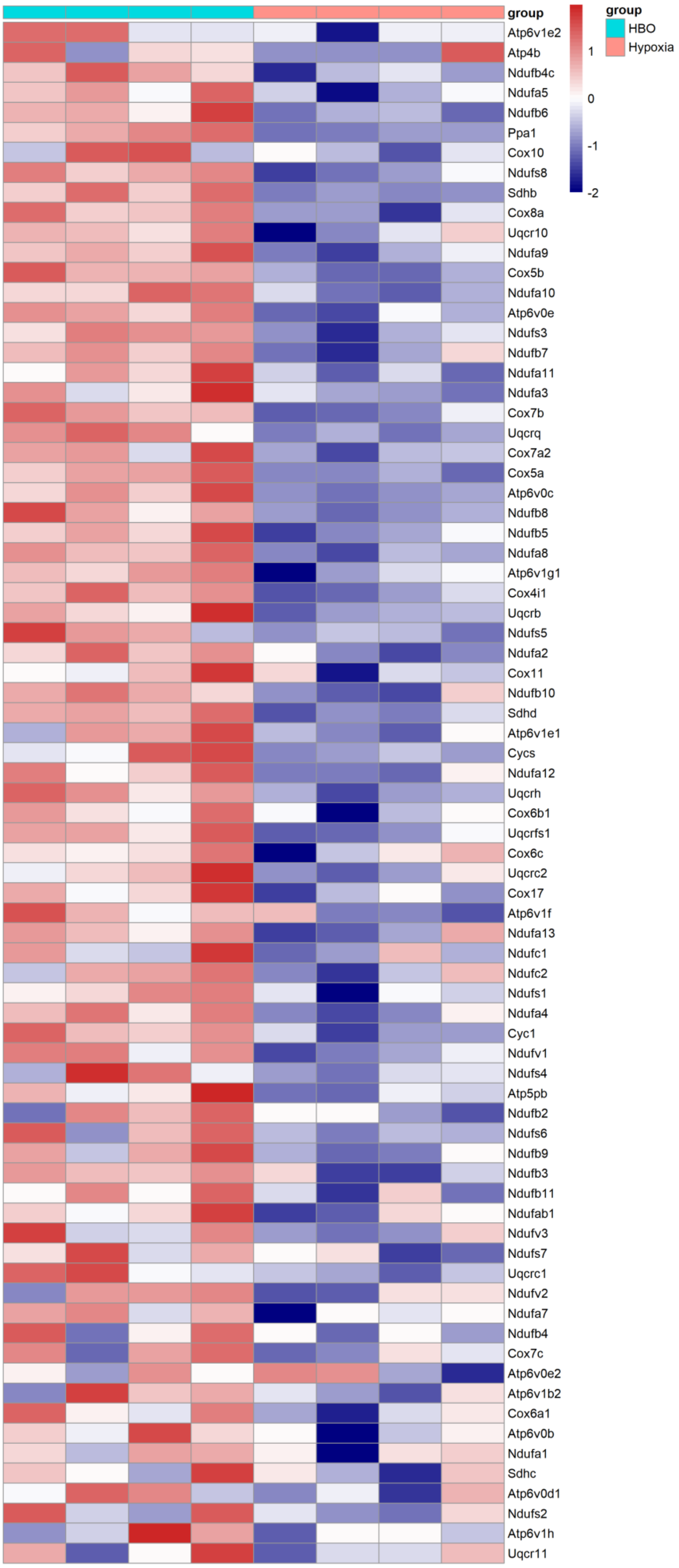

Figure S3 Heatmap of genes that were significantly altered in oxidative phosphorylation (OXPHOS).

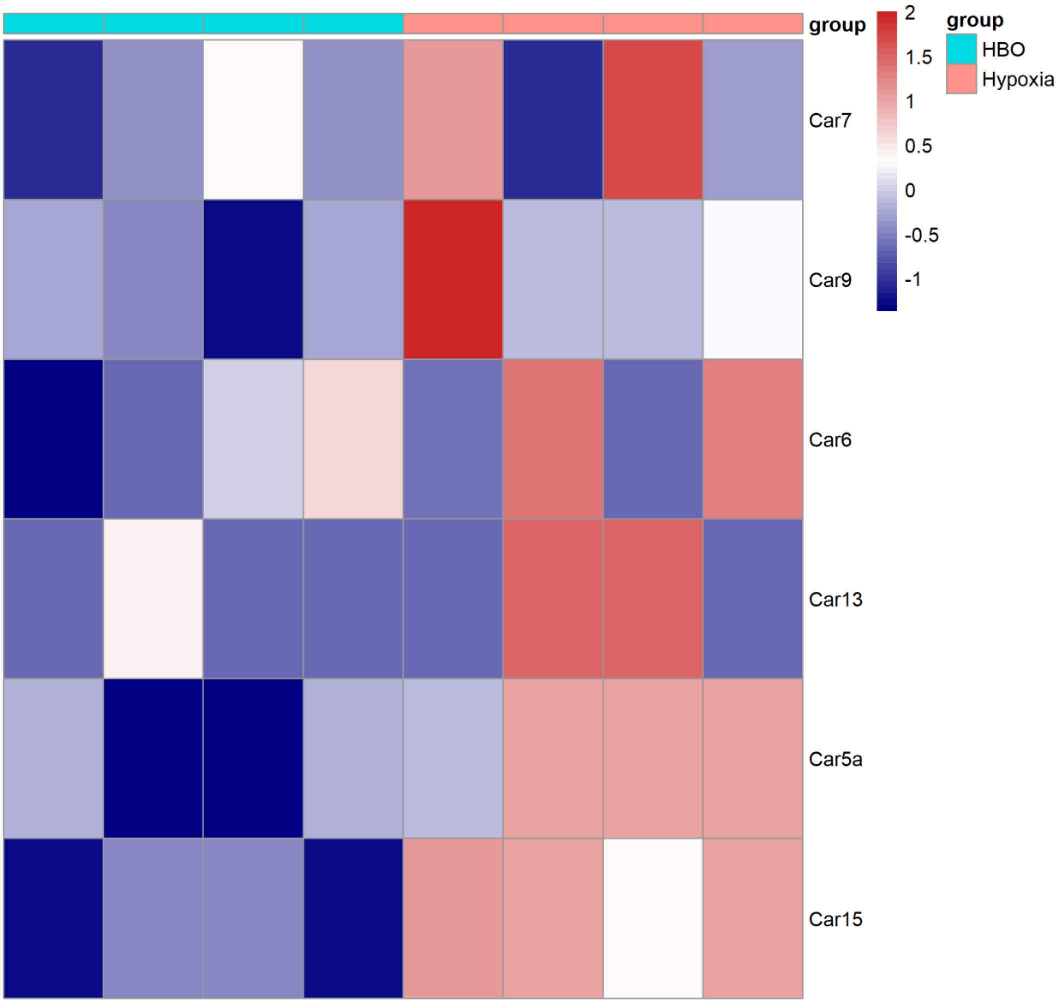

Figure S4 Heatmap of genes that were significantly altered in the nitrogen metabolism.

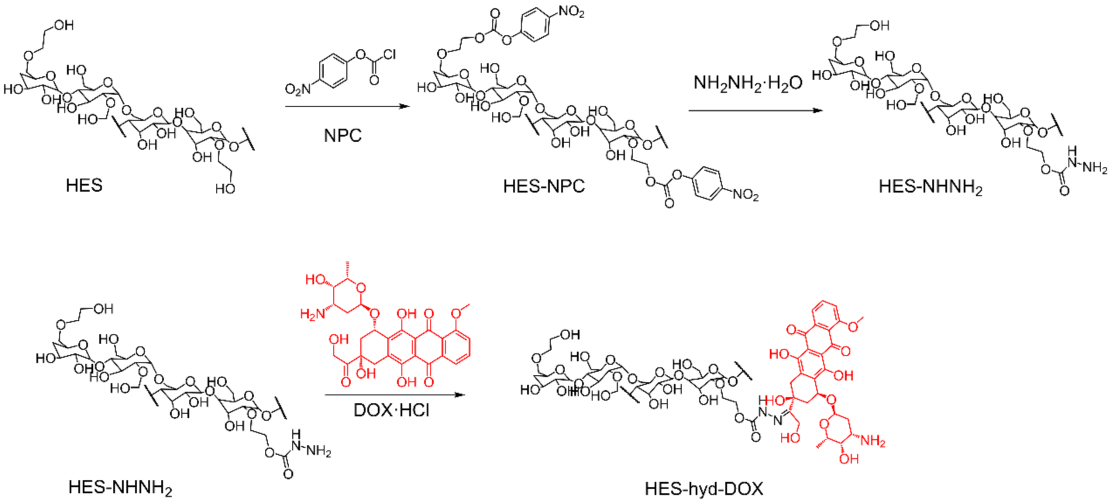

Figure S5 Synthetic scheme for HES-hyd-DOX.

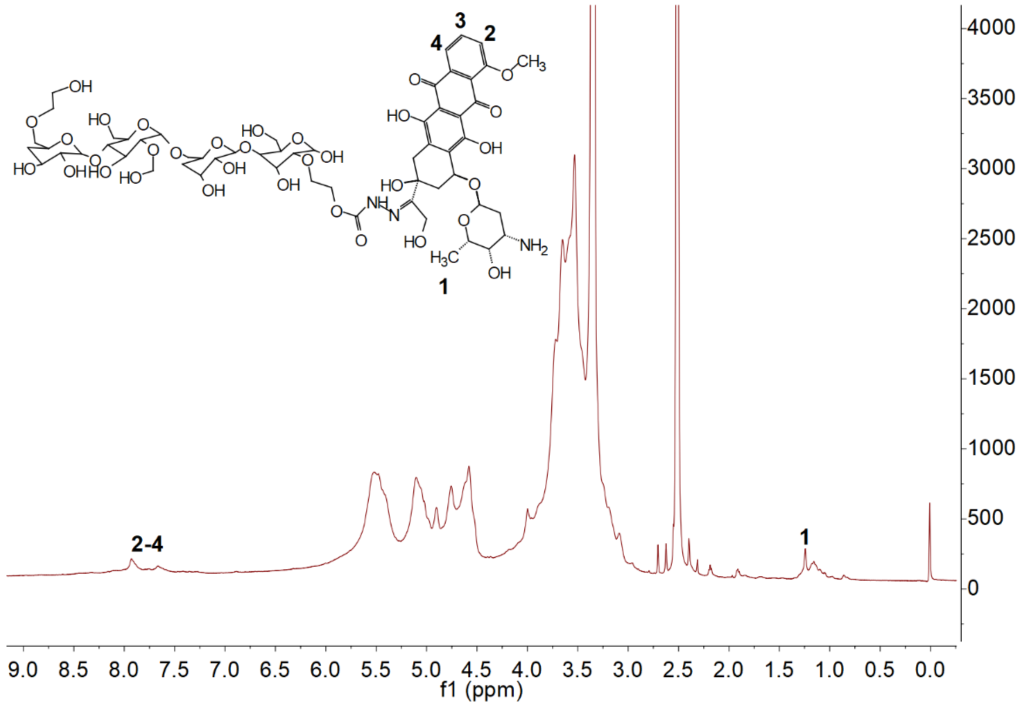

Figure S6 ¹H NMR spectrum of HES-hyd-DOX.

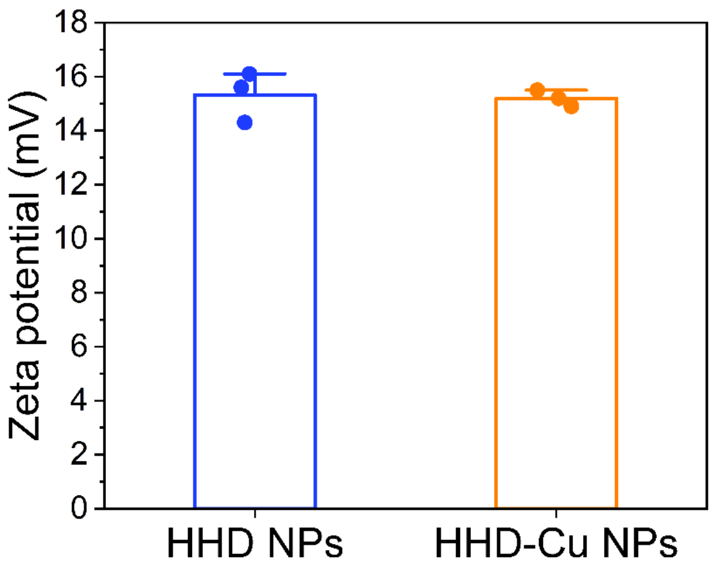

Figure S7 Zeta potential of HHD NPs and HHD-Cu NPs.

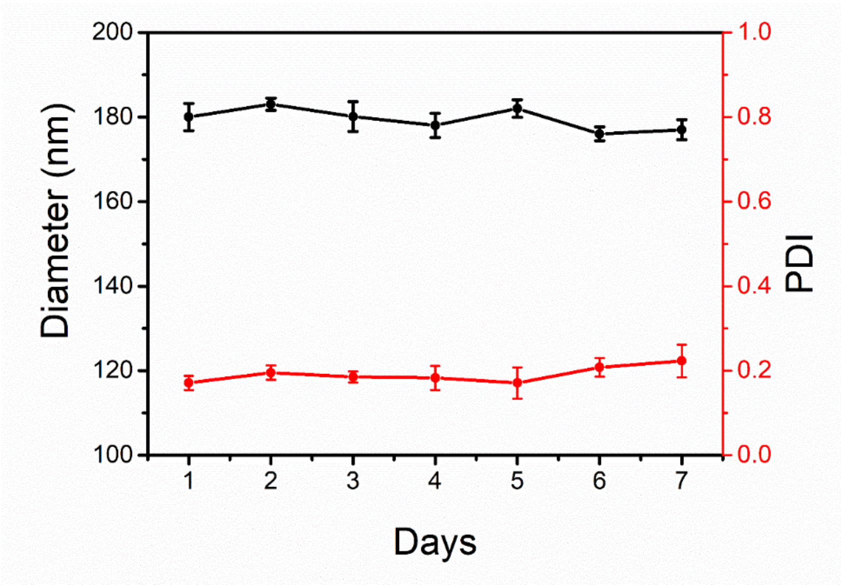

Figure S8 Seven days stability test of HHD-Cu NPs.

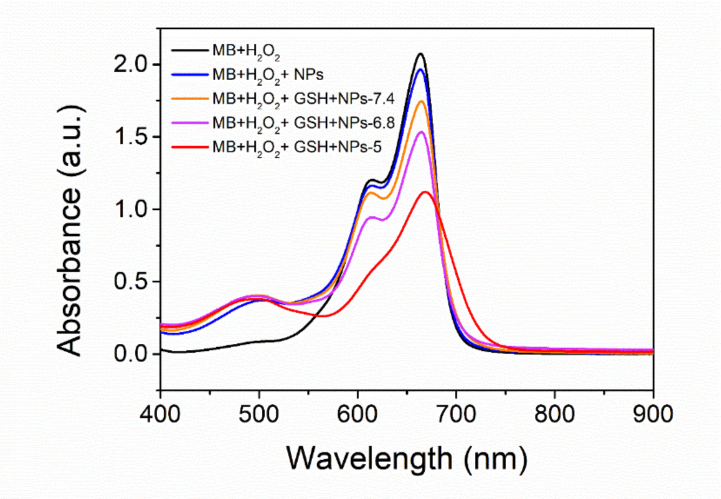

Figure S9 MB assay detecting hydroxyl radicals generated by HHD-Cu NPs under different pH conditions.

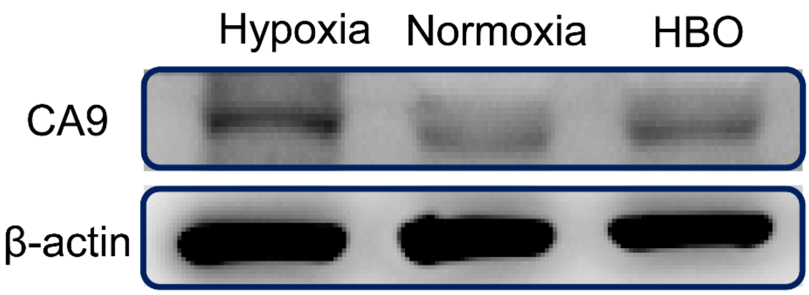

Figure S10 Expressions of CA9 proteins in H22 cells under different oxygen

concentrations.

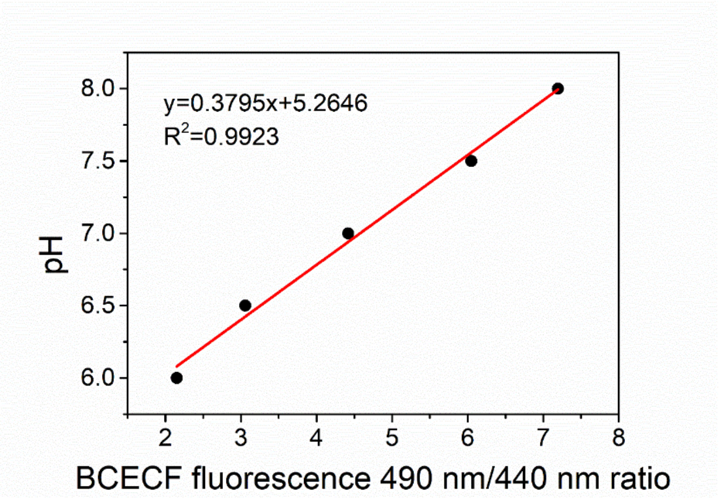

Figure S11 Standard curve for intracellular pH measurement using BCECF.

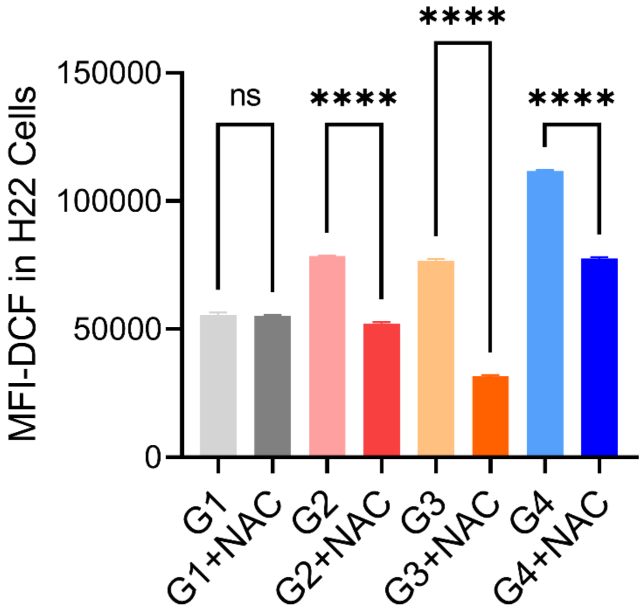

Figure S12 Intracellular ROS levels in H22 cells after various treatments. G1, Hypoxia; G2, HHD-Cu NPs; G3, HBO; G4, HHD-Cu NPs + HBO. Statistical significance was calculated by One-way ANOVA followed by post hoc Tukey's test and represented as the mean ±SEM. *p* values: * $p < 0.05$, ** $p < 0.01$, *** $p < 0.001$, **** $p < 0.0001$, ns stands for not significant.

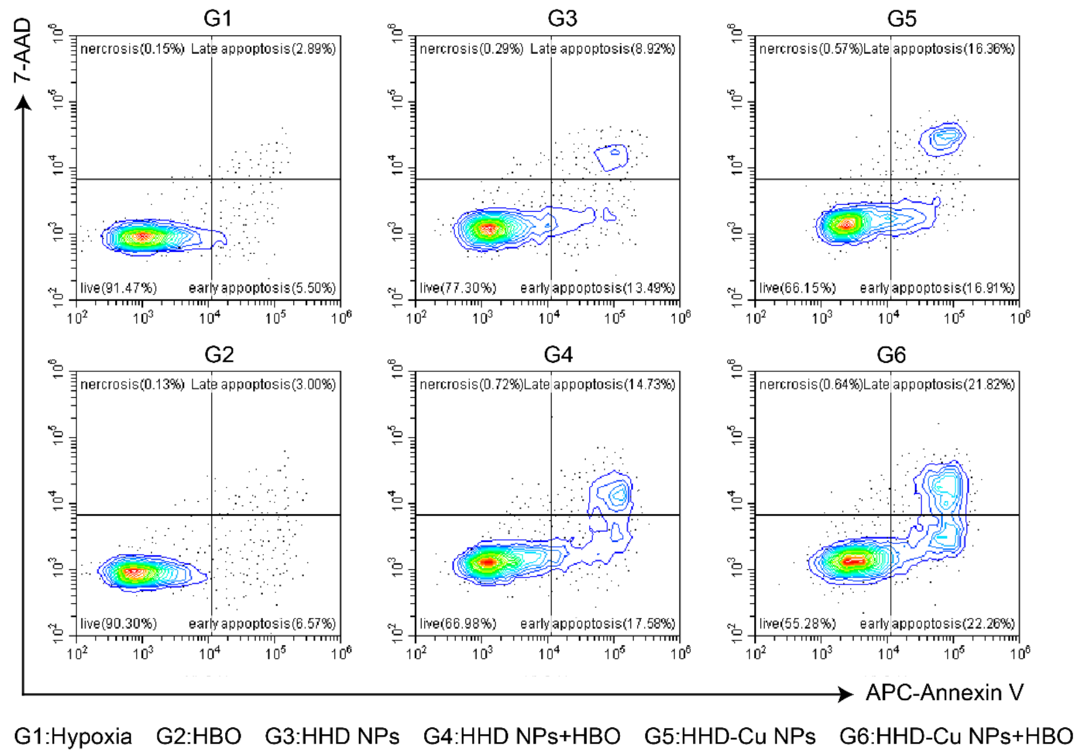

Figure S13 Flow cytometry gating strategy for apoptosis testing in H22 cells after different treatments.

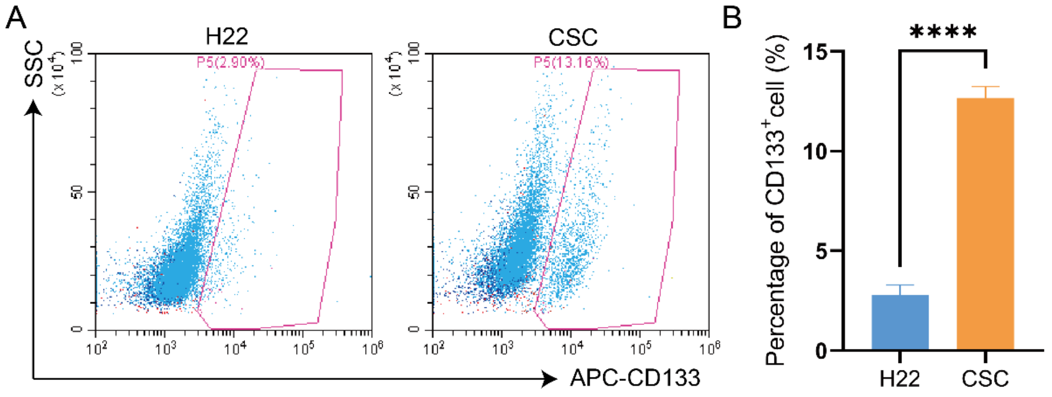

Figure S14 (A) Flow cytometry gating strategy for $CD133^{+}$ cells of H22 cells and CSCs. (B) Comparison of $CD133^{+}$ cells in H22 cells and CSCs. Statistical significance was calculated by unpaired two-sided Student's t test and represented as the mean ±SEM. p values: * $p < 0.05$, ** $p < 0.01$, *** $p < 0.001$, **** $p < 0.0001$, ns stands for not significant.

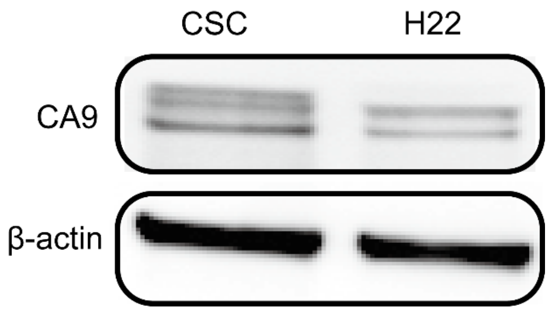

Figure S15 Differential expression of CA9 in H22 cells and CSCs.

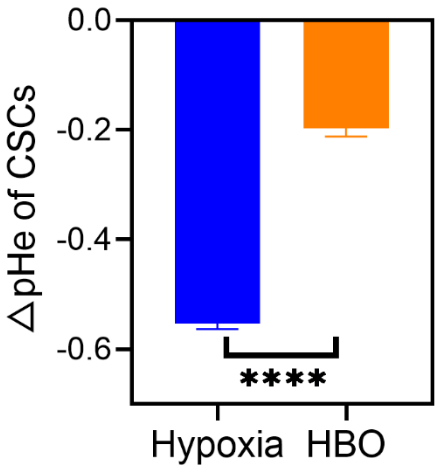

Figure S16 Changes in extracellular pH of CSCs. Statistical significance was calculated by unpaired two-sided Student's t test and represented as the mean ±SEM. *p* values: * $p < 0.05$, ** $p < 0.01$, *** $p < 0.001$, **** $p < 0.0001$, ns stands for not significant.

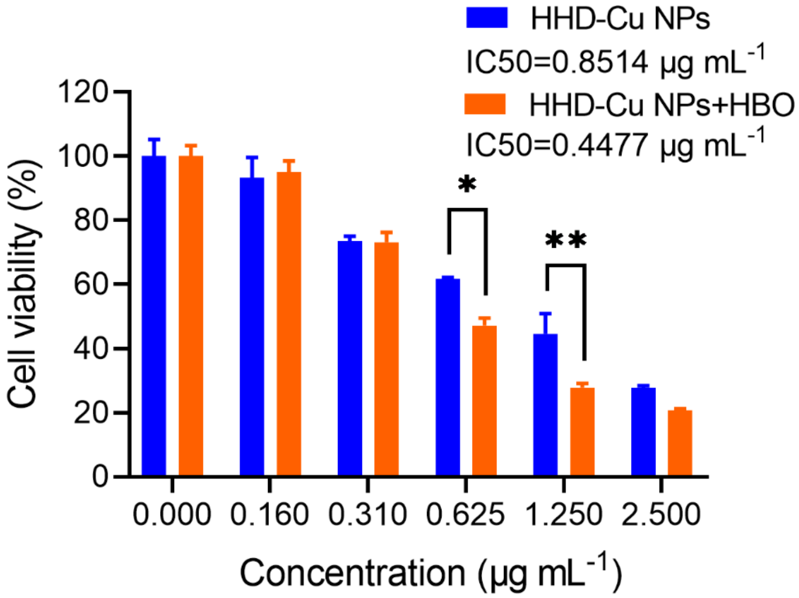

Figure S17 Influence of HBO on cytotoxicity of HHD-Cu NPs against H22 CSCs. Statistical significance was calculated by unpaired two-sided Student's t test and represented as the mean ±SEM. * p < 0.05, ** p < 0.01, *** p < 0.001, **** p < 0.0001, ns stands for not significant.

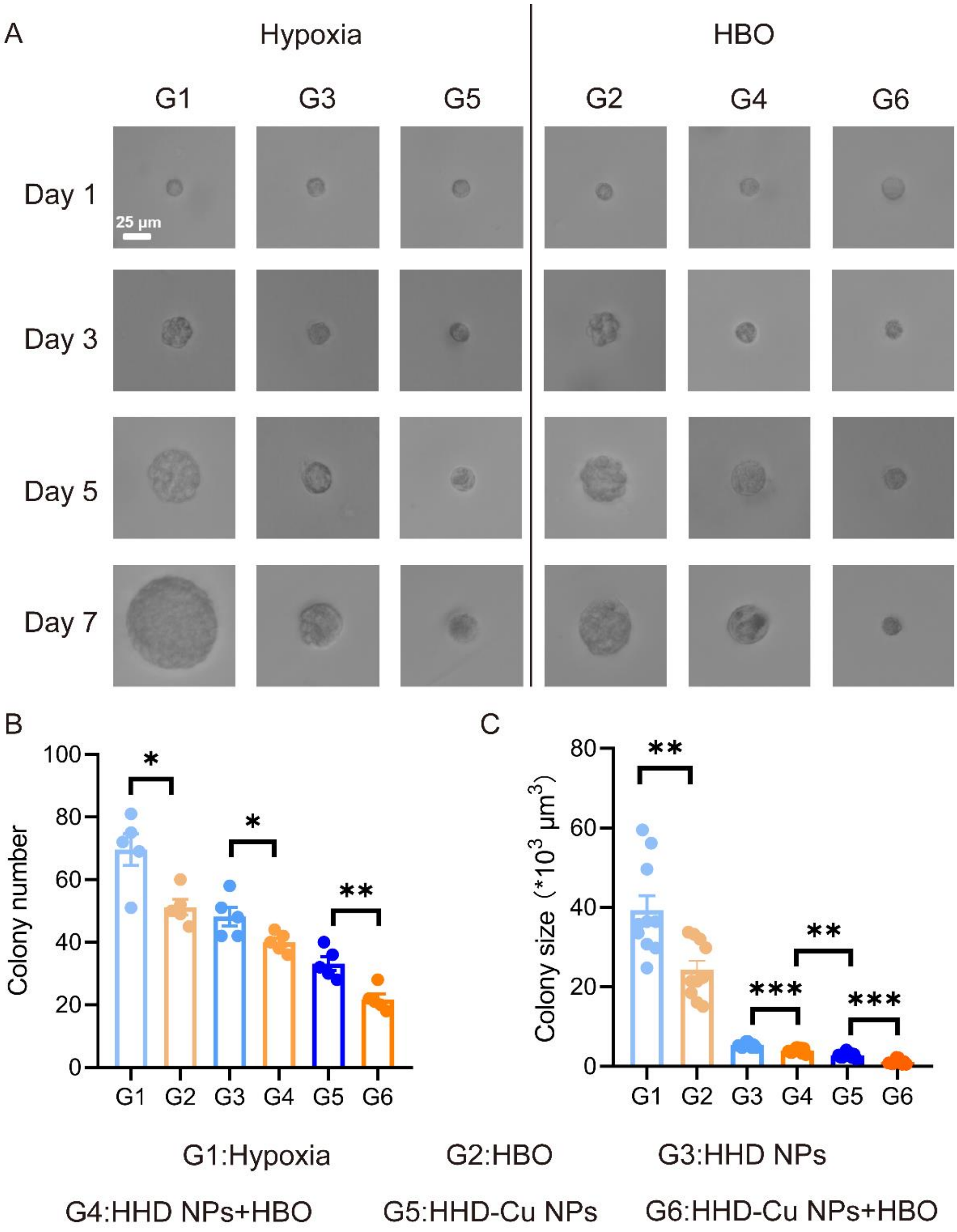

Figure S18 (A) Inhibition of H22 cell spheroid formation and growth in 3D fibrin gels by different treatments. The colony number (B) (n = 5 independent replicates) and colony size (C**)** (n = 10 independent replicates) at day 7. Statistical significance was calculated by unpaired two-sided Student's t test and represented as the mean ±SEM. p values: * $p < 0.05$, ** $p < 0.01$, *** $p < 0.001$, **** $p < 0.0001$, ns stands for not significant.

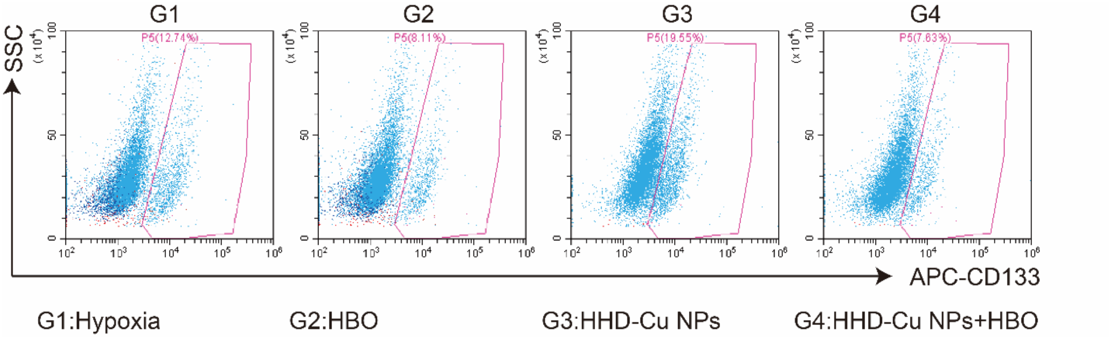

Figure S19 Flow cytometry gating strategy for $CD133^+$ cells after various treatments.

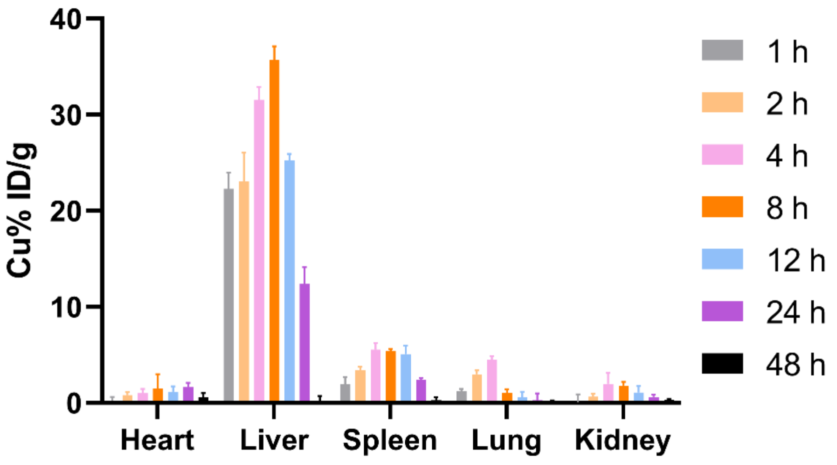

Figure S20 The biodistribution of HHD-Cu NPs (n=3 mice).

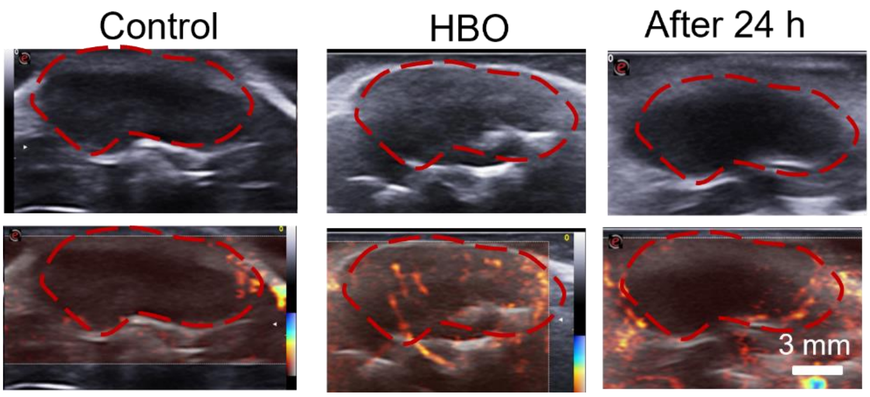

Figure S21 HBO increases blood flow within H22 tumors.

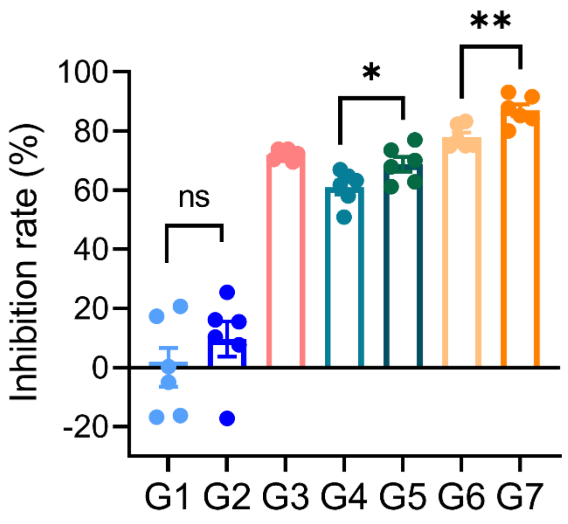

Figure S22 Calculation of *in vivo* tumor inhibition rate of different treatment groups (n=6). Data are expressed as mean ± SEM. Statistical significance was calculated by unpaired two-sided Student's t test and represented as the mean ± SD. * $p < 0.05$, ** $p < 0.01$, *** $p < 0.001$, **** $p < 0.0001$, ns stands for not significant. The groups are saline (G1), HBO (G2), DOX (G3), HHD NPs (G4), HHD NPs + HBO (G5), HHD-Cu NPs (G6) and HHD-Cu NPs + HBO (G7).

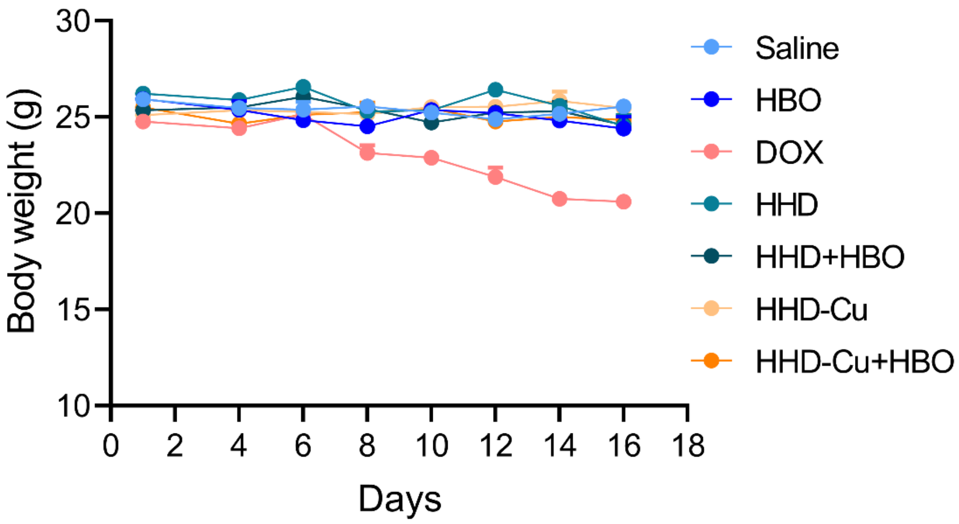

Figure S23 Body weight of various treatments (n=6 mice).

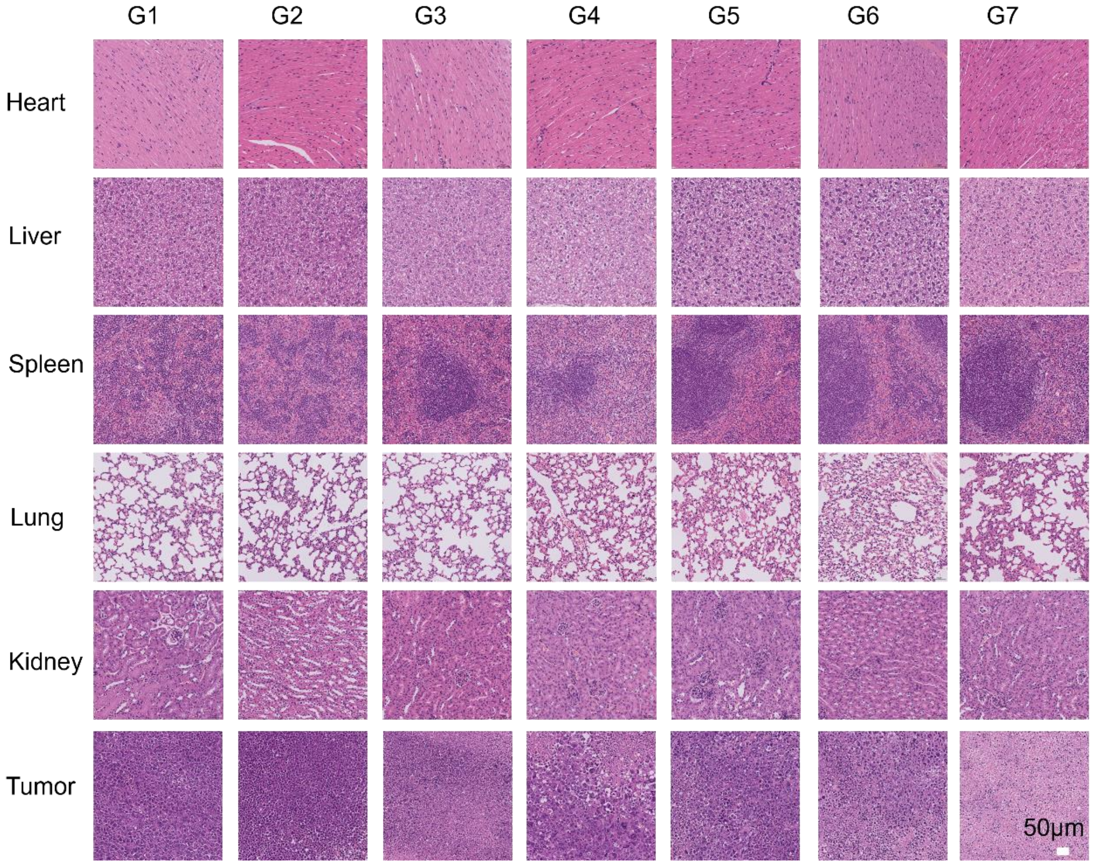

Figure S24 H&E staining of major organs and tumor of each group, scale bar: 50 μm. G1, Saline; G2, HBO; G3, DOX; G4, HHD NPs; G5, HHD NPs + HBO; G6, HHD-Cu NPs; G7, HHD-Cu NPs + HBO.

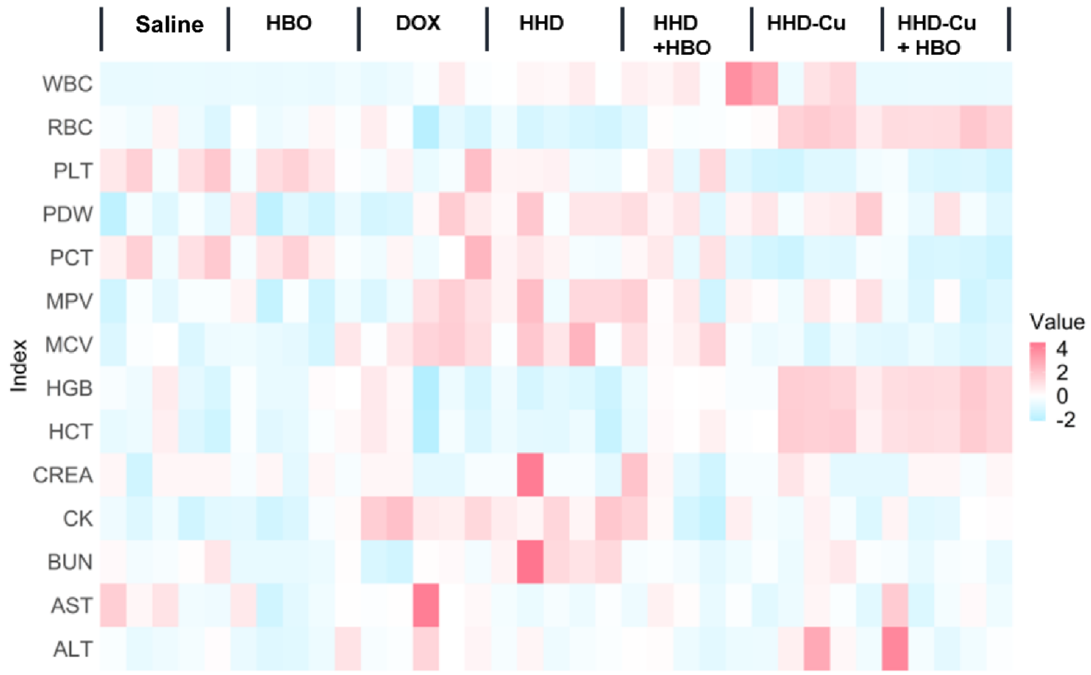

Figure S25 Heatmap of blood biochemical and blood routine in mice of different treatments (n = 5 mice).

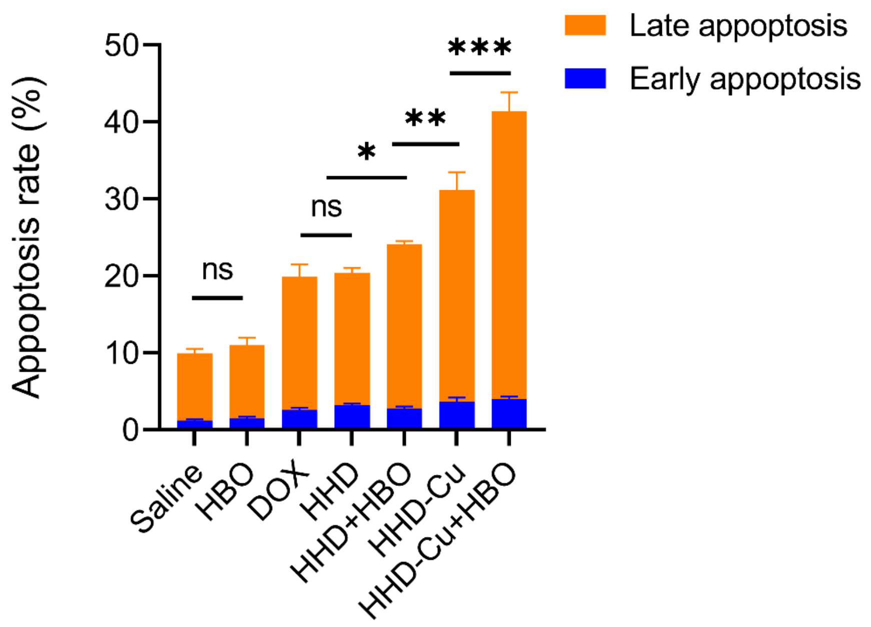

Figure S26 Assessment of apoptosis in tumor tissues after different treatments (n=4 mice). Statistical significance was calculated by unpaired two-sided Student's t test and represented as the mean ±SEM. p values: * $p < 0.05$, ** $p < 0.01$, *** $p < 0.001$, **** $p < 0.0001$, ns stands for not significant.

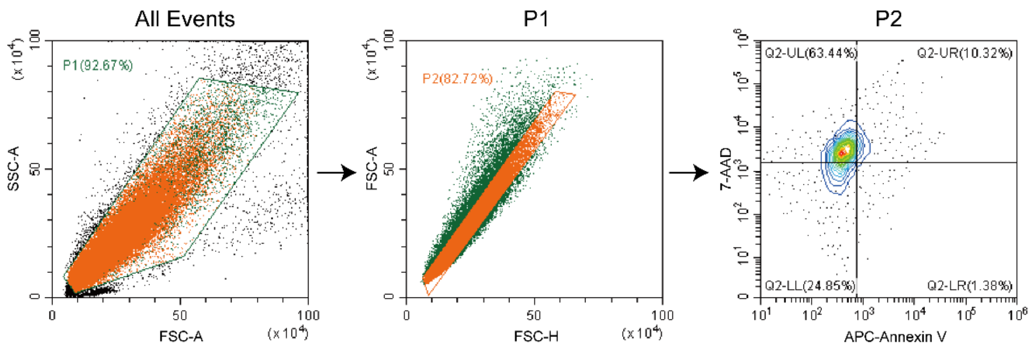

Figure S27 Flow cytometry gating strategy for apoptosis detection in single-cell suspension after digestion of subcutaneous tumors.

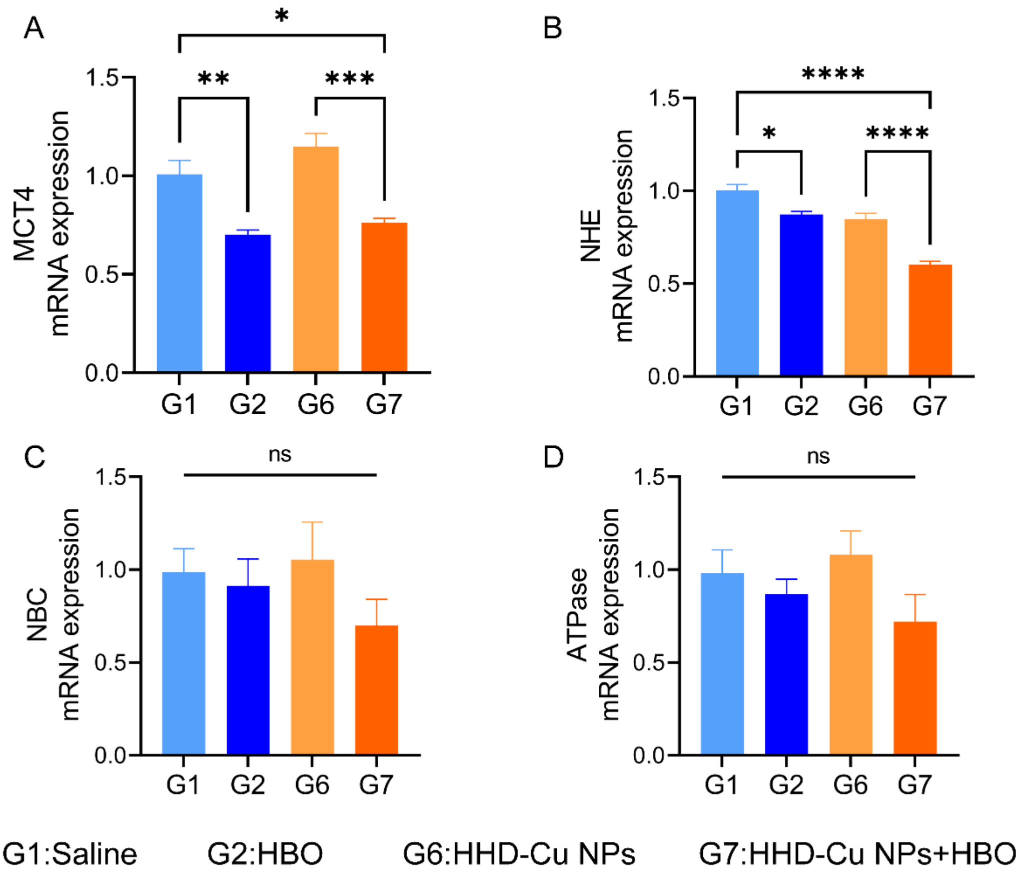

Figure S28 Transcriptional expression of genes related to pH regulatory pathways in H22 subcutaneous tumors. MCT4 (A), NHE (B), NBC (C) and V-ATPase (D) mRNA as normalized to β-actin of tumor cells after various treatments. Statistical significance was

calculated by unpaired two-sided Student's t test and represented as the mean ±SEM. *p* values: * $p < 0.05$, ** $p < 0.01$, *** $p < 0.001$, **** $p < 0.0001$, ns stands for not significant.

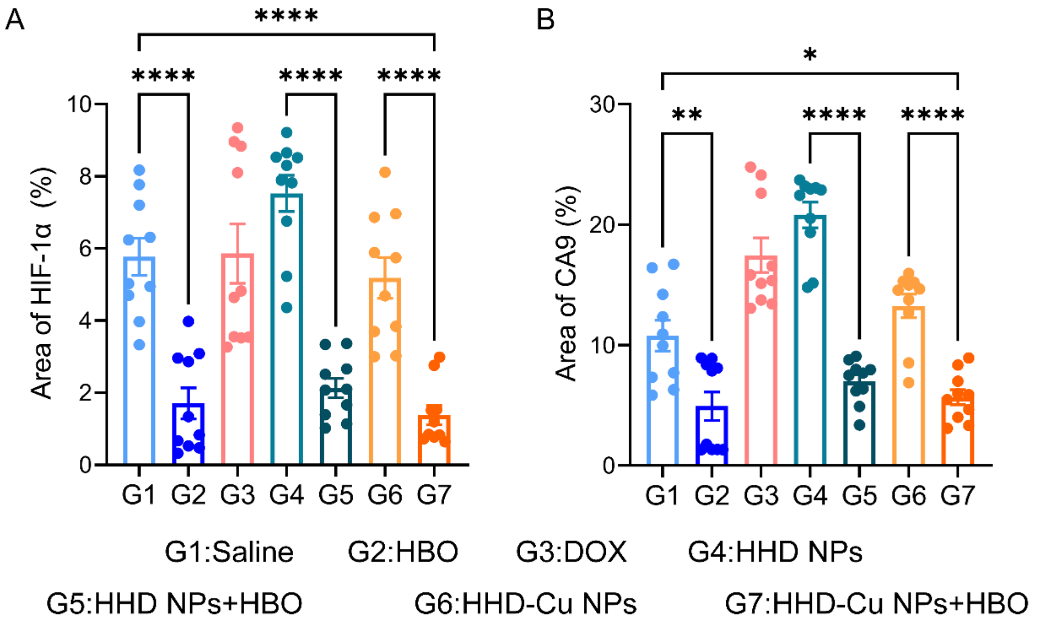

Figure S29 Percentage of the area of (A) HIF-1α and (B) CA9 (n = 10 independent replicates). Statistical significance was calculated by One-way ANOVA followed by post hoc Tukey's test and represented as the mean ±SD. *p* values: * $p < 0.05$, ** $p < 0.01$, *** $p < 0.001$, **** $p < 0.0001$, ns stands for not significant.

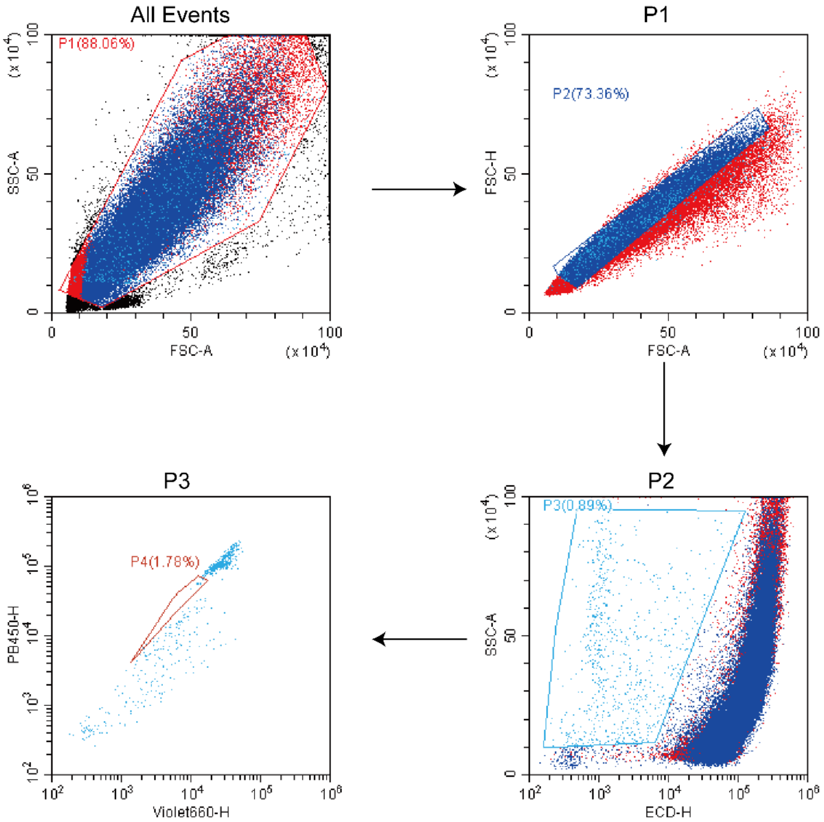

Figure S30 Flow cytometry gating strategy for SP cells.

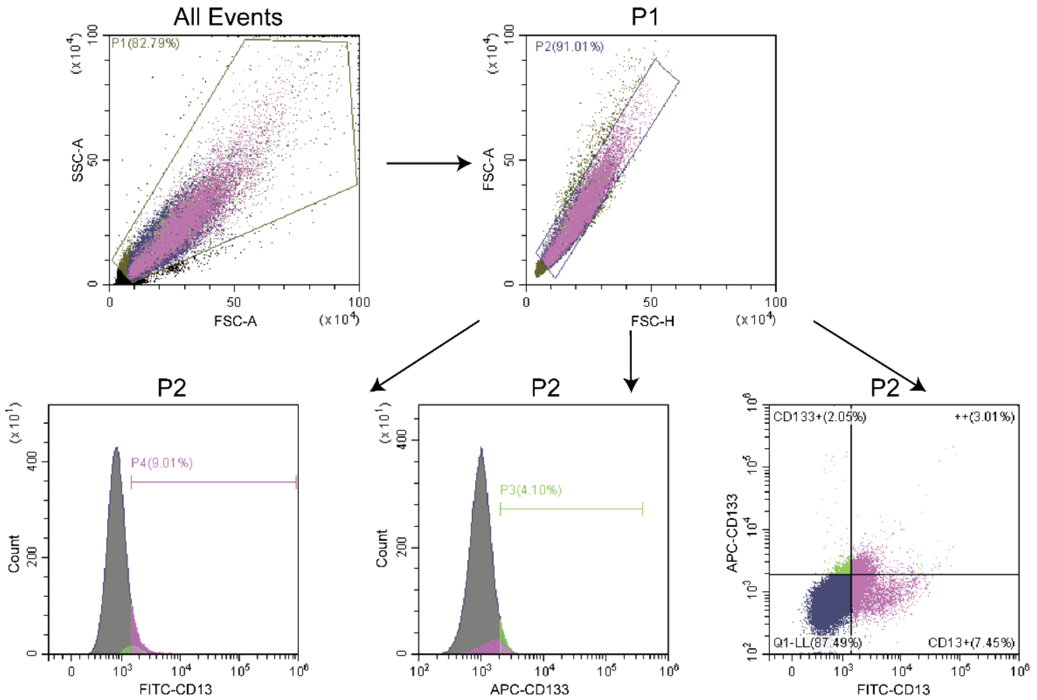

Figure S31 Flow cytometry gating strategy for $CD13^{+}CD133^{+}$ CSCs.

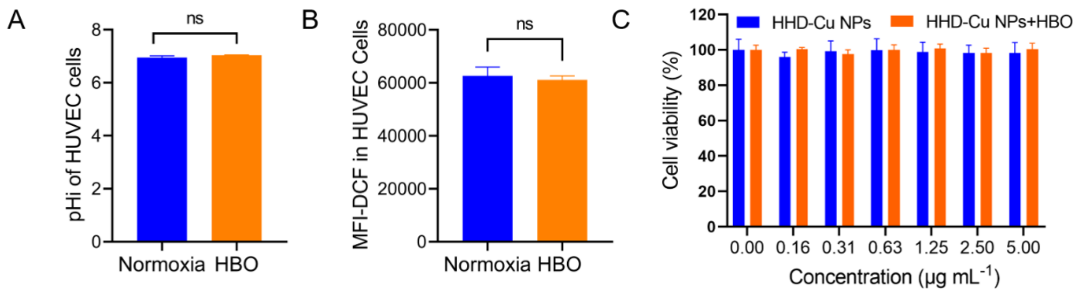

Figure S32 Cytotoxicity of HBO and HHD-Cu NPs on HUVEC cell lines. (A) pHi and (B) ROS level within HUVEC cells after HBO treatment. (C) Cell viability assay of HUVEC after HHD-Cu NPs treatment. Statistical significance was calculated by unpaired two-sided Student's t test and represented as the mean ±SEM. *p* values: * $p < 0.05$, ** $p < 0.01$, *** $p < 0.001$, **** $p < 0.0001$, ns stands for not significant.

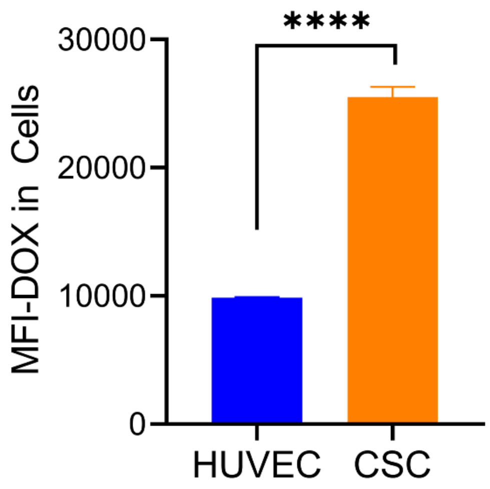

Figure S33 Mean fluorescence intensity of DOX in HUVEC and CSC within 2 h after HHD-Cu NPs treatment. Statistical significance was calculated by unpaired two-sided Student's t test and represented as the mean ±SEM. *p* values: * $p < 0.05$, ** $p < 0.01$, *** $p < 0.001$, **** $p < 0.0001$, ns stands for not significant.

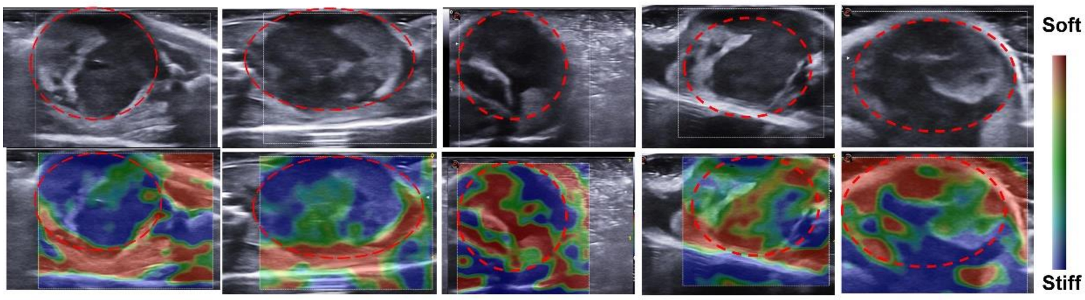

Figure S34 Ultrasound elastography images of subcutaneous H22 tumors at different time points.

Table S1 Key pharmacokinetic parameters of HHD-Cu NPs and HBO combined with HHD-Cu NPs.

	**HHD-Cu NPs**	**HHD-Cu NPs + HBO**
**C_{max} (μg mL^{-1})**	5.65	7.67
**$t_{1/2}$ (h)**	1.54	2.38
**CL (L h^{-1} kg^{-1})**	0.12	0.091
**$AUC_{(0\to t)}$ (mg h L^{-1})**	16.5	18.81

C_{max}, peak concentration; $t_{1/2}$, half-life time; CL, plasma clearance; AUC, area under the curve. The data were obtained using a two-compartment model.
